# Evaluating the efficacy of basiliximab versus no induction in low-immunological-risk kidney transplant recipients: a propensity score matched analysis

**DOI:** 10.1080/0886022X.2025.2460729

**Published:** 2025-02-20

**Authors:** Dahao Zhang, Jiqiu Wen, Jianhui Dong, Rong Ma, Shijian Li, Jihua Wu, Ning Wen, Zhiying Lei, Haibin Li, Jun Yin, Xuyong Sun

**Affiliations:** Institute of Transplantation Medicine, The Second Affiliated Hospital of Guangxi Medical University, Guangxi Clinical Research Center for Organ Transplantation, Guangxi Key Laboratory of Organ Donation and Transplantation, Nanning, China

**Keywords:** Kidney transplantation, induction therapy, no induction, low-immunological-risk, infection, propensity score matching

## Abstract

**Background:**

The optimal use of induction therapy in low-immunological-risk kidney transplant recipients (KTRs) remains uncertain. While Basiliximab (BSX) is widely utilized, its comparative outcomes with no induction therapy require further evaluation.

**Method:**

This single-center retrospective cohort study included 182 low-immunological-risk KTRs who underwent transplantation between January 2022 and March 2023. Patients were assigned to either no induction (*n* = 41) or BSX induction (*n* = 141) groups. Propensity score matching (PSM) minimized selection bias and controlled for confounding factors. Primary outcomes included the incidence of first acute rejection (AR) within 12 months, while secondary outcomes encompassed graft function, infection rates, and adverse events.

**Result:**

After 12 months, the cumulative AR incidence was comparable between groups (*p* = 0.46). The no induction group demonstrated superior renal function, with consistently higher estimated glomerular filtration rates (eGFR) at early postoperative intervals. Additionally, this group exhibited reduced infection-related hospitalizations (respiratory infections: 7.32 vs. 29.1%, *p* = 0.008) and hematological complications (thrombocytopenia: 0.00% vs. 12.8%, *p* = 0.014). Mortality and graft loss rates were similar between groups.

**Conclusion:**

In low-immunological-risk KTRs, no induction therapy achieves comparable AR prevention and renal function outcomes to BSX while reducing infection and hematological complications. These findings challenge the necessity of universal induction therapy in this population and support a personalized approach to immunosuppression protocols.

## Graphical Abstract

**Figure:**
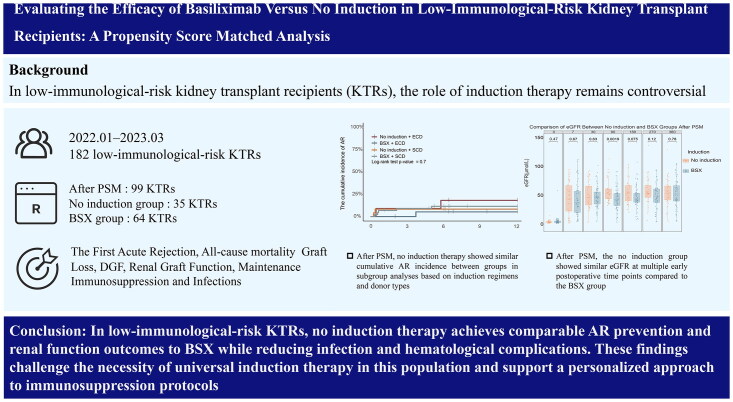

## Introduction

Kidney transplantation is the optimal treatment for end-stage renal disease [1,2], offering higher survival rates, better quality of life, and lower medical costs compared to dialysis treatment [3]. Induction therapy is crucial in immunosuppression, providing strong immunosuppression when the risk of acute rejection is highest [4]. According to the 2022 OPTN/SRTR report [5], 92.1% of kidney transplant recipients (KTRs) in the United States received biological induction agents, with Basiliximab (BSX) being the most commonly used. Additionally, 92.9% were discharged on a maintenance regimen consisting of tacrolimus (TAC) and mycophenolic acid (MPA).

Basiliximab, a chimeric monoclonal antibody specifically targeting the interleukin-2 receptor α chain (CD25), achieves immunosuppression through selective inhibition of T lymphocyte activation [6]. Its efficacy was established through randomized controlled trials (RCTs) incorporated in the 2009 KDIGO guidelines, which demonstrated reduced acute rejection rates without significant increases in infection risk [7,8]. Additionally, BSX shows a favorable long-term safety profile, with low incidences of malignancy and post-transplant lymphoproliferative disorders [9]. These characteristics, combined with its non-lymphocyte-depleting mechanism and standardized dosing protocol, have positioned BSX as a preferred option for low-immunological-risk KTRs.

However, the evidence supporting BSX induction warrants reexamination in the context of modern immunosuppression protocols. Most foundational RCTs were conducted in an era when cyclosporine (CsA) and azathioprine (AZA) constituted standard maintenance therapy. Contemporary practice has evolved to favor triple maintenance immunosuppression with TAC/MPA/Pred, which has fundamentally altered the risk-benefit equation [10]. Recent studies demonstrate that TAC/MPA-based regimens alone reduce acute rejection rates from 40–50% to 10–15% compared to historical CsA/AZA protocols [11,12]. In this context, the additional benefit of antibody induction in low-immunological-risk KTRs appears modest, providing only a 1–4% absolute risk reduction [13,14].

Despite widespread BSX utilization, there remains a critical gap in the evidence base: no large-scale, multicenter, prospective, randomized controlled trial has evaluated induction strategies specifically in low-risk KTRs receiving modern TAC-based maintenance regimens. This knowledge gap raises important questions about the necessity of BSX induction in this population. Therefore, our study aims to evaluate the safety and efficacy of foregoing induction therapy in low-immunological-risk KTRs maintained on contemporary triple-regimen immunosuppression (TAC/MPA/Pred).

## Method

### Study design

This retrospective cohort study evaluated the safety and efficacy of no induction therapy in low-immunological-risk kidney transplant recipients (KTRs). Recipients who underwent their first allogeneic kidney transplantation from January 2022 to March 2023 at the Second Affiliated Hospital of Guangxi Medical University were included. We compared the cumulative incidence of first acute rejection within 12 months, all-cause mortality, and graft loss (death-censored) between the two groups. The impact of different induction regimens on acute rejection was analyzed using a multivariate Cox model. Pairwise comparisons were performed between the two groups, and propensity score matching (PSM) was used to adjust for confounding factors. This study was approved by the Clinical Research Ethics Committee of the Second Affiliated Hospital of Guangxi Medical University under approval number: 2023-KY(0929). Informed consent was waived due to the retrospective nature of the study. All clinical and research activities strictly adhered to the principles of the Istanbul Declaration

### Inclusion and exclusion criteria

Inclusion criteria were: recipients aged 18–65 years undergoing their first allogeneic kidney transplantation; negative HLA-I and II antibodies; negative lymphocyte cross-match; cold ischemia time ≤12 h; maintenance immunosuppression with TAC/MPA/Pred at discharge; and negative panel reactive antibody (PRA).

Exclusion criteria were: follow-up less than 6 months; receiving other induction regimens; incomplete clinical data; multi-organ transplantation; donor after cardiac death (DCD); ABO-incompatible transplantation; active systemic infection before transplantation; risk of malignant tumor recurrence; history of substance abuse or mental disorders; HIV seropositivity; positive hepatitis B surface antigen (HBsAg) or hepatitis C virus DNA (HCV-DNA); contraindications to regimens; allergy or severe neutropenia; and not receiving the full 40 mg dose of BSX.

### Study population

A total of 182 kidney transplant recipients (KTRs) were included: 41 with no induction therapy (No induction group) and 141 with basiliximab induction (BSX group). The pre-transplant cross-matching test was negative. Laboratory detection methods included sequence-specific oligonucleotide probe hybridization (SSO) to identify HLA types in patients. Six HLA SSO typing kits purchased from IMMUCOR were used to detect the HLA types of recipients, including HLA-A, HLA-B, HLA-DRB1, HLA-C, HLA-DQA+B, and HLA-DPA+B.

All recipients underwent their first kidney transplantation and received maintenance immunosuppression with TAC/MPA/Pred. Preoperative tests for HLA-I and HLA-II antibodies and PRA were negative, and cold ischemia time was less than 12 h. All recipients had ABO blood type compatibility and received kidneys from DBD. Most recipients (85.7%) had ≤4 HLA mismatches, indicating a low-immunological-risk [15].

Protocol biopsies were not performed. Kidney biopsies were conducted under ultrasound guidance when clinically indicated, such as delayed graft function (DGF), oliguria or anuria, suspected rejection signs (fever, decreased urine output, graft swelling and tenderness, rising serum creatinine), unexplained serum creatinine increase over 30% of baseline, persistent abnormal urinalysis (proteinuria >1 g/24 h, >100 red blood cells per high-power field), or imaging showing a renal artery resistance index (RI) >0.8 on Doppler ultrasound. If a biopsy was not performed but treatment was effective, a diagnosis of acute rejection was made clinically based on the patient’s response to therapy and clinical presentation.

### Maintenance immunosuppression

All recipients received a standard triple-drug maintenance immunosuppressive regimen consisting of tacrolimus, mycophenolic acid (MPA), and prednisone (TAC/MPA/Pred). Postoperative follow-up was conducted weekly during the first month, biweekly from months 2 to 3, monthly from months 3 to 6, and every three months from months 6 to 12.

Tacrolimus (TAC) was initiated at a dosage of 0.05–0.15 mg/(kg·d) [16], with target trough levels maintained between 8 and 15 ng/ml for the first six months post-transplantation.

Prednisone (Pred), a corticosteroid hormone, was started at 30 mg/day within 24 h after surgery, then gradually tapered to 10–15 mg/day by postoperative day 30, and continued as long-term maintenance therapy. Early withdrawal or complete avoidance of prednisone is not currently recommended [17].

Mycophenolic Acid (MPA) was introduced prior to or within 24 h following transplantation and maintained throughout the follow-up period [18]. The recommended dose of mycophenolate mofetil (MMF) was 1.5–2.0 g/day for recipients weighing ≥70 kg, 1.5 g/day for those weighing 50–69 kg, and 1.0 g/day for those weighing < 50 kg, administered in two divided doses. The starting dose of enteric-coated mycophenolate sodium (EC-MPS) was 360–720 mg, given orally twice daily.

### Prophylaxis against infection

All recipients received standard prophylaxis with ganciclovir for cytomegalovirus (CMV) infection prevention and sulfamethoxazole-trimethoprim (TMP-SMZ) for *Pneumocystis jirovecii* pneumonia (PJP) prophylaxis [19]. For recipients of CMV-seropositive donors, enhanced surveillance was implemented, and the duration of prophylaxis was prolonged based on clinical indications.

### Efficacy endpoint

The primary outcome was the incidence of first acute rejection within 12 months post-transplantation. This included biopsy-proven acute rejection (BPAR) graded according to the 2019 Banff classification [20], including borderline changes meeting its criteria, and clinically diagnosed acute rejection. Clinically diagnosed acute rejection is defined by meeting the following criteria, along with an effective response to anti-rejection therapy: (1) Clinical manifestations, including sudden reduction in urine output, gradual weight gain over several days, unexplained hypertension, or fever of unknown origin; (2) Physical examination findings, such as swelling and tenderness of the transplant kidney on palpation or audible vascular bruits on auscultation; (3) Auxiliary examinations, including increased serum creatinine during recovery, proteinuria and hematuria on urinalysis, Doppler ultrasound showing resistive index (RI) >0.8, or positive HLA-I or HLA-II antibodies.

Secondary outcomes were estimated glomerular filtration rate (eGFR), tacrolimus trough levels, recipient cumulative survival, cumulative incidence of graft loss (death-censored), incidence and duration of delayed graft function (DGF), modifications to the standard triple immunosuppressive maintenance regimen, and incidence of postoperative adverse events (including hematological disorders such as thrombocytopenia, leukopenia, and infections requiring hospitalization). DGF was defined as the need for dialysis within the first week after kidney transplantation due to non-recovery of graft function [21]. Extended criteria donors (ECD) were defined as those aged ≥60 years, or 50–59 years meeting two or more of the following criteria: (1) cerebrovascular accident as cause of death; (2) history of hypertension; (3) pre-donation serum creatinine >132.6 μmol/L [22].

### Data collection and definition

Clinical information was independently retrieved by two observers from the electronic medical record system. Baseline characteristics included recipient age, sex, body mass index (BMI), primary kidney disease, number of HLA mismatches, donor type, and induction therapy regimens. Laboratory data collected during post-transplant follow-up included eGFR, tacrolimus trough levels, time to first acute rejection episode, recipient survival, graft loss, and occurrence and duration of DGF. Adverse events included leukopenia, thrombocytopenia, and infections requiring hospitalization confirmed by culture or next-generation sequencing (NGS).

### Statistical analysis

This retrospective cohort study was conducted using R software (version 4.3.2) and the ggplot2 package (version 3.5.0). Variables with >10% missing data were excluded. For variables with <10% missing data, such as eGFR (5.95%) and tacrolimus trough levels (7.1%), missing values were imputed using predictive mean matching (PMM) with the ‘mice’ package (version 3.16.0). In the process of imputing missing values for eGFR, we collected variables closely related to eGFR, such as CREA, Cystatin C, and HCO3 (Figure S1(A)), and included them in the imputation process to enhance the accuracy of the imputed datasets. Using the ‘Mice’ package in R, we performed multiple imputation with the Predictive Mean Matching (PMM) algorithm, generating 20 datasets with up to 50 iterations for each. However, as there is currently no automatic method to identify the best imputed dataset, we assessed the validity of the imputation results by comparing the distribution curves before and after imputation (Figure S1(B,C)). The multiple imputation process for TAC was similar to that of eGFR.

Normally distributed continuous variables were reported as mean ± standard deviation (SD) and compared between groups using one-way analysis of variance (ANOVA). Non-normally distributed continuous variables were presented as median (interquartile range, IQR) and compared using the Mann–Whitney *U* test. Categorical variables were expressed as frequencies and percentages and compared using the chi-square test or Fisher’s exact test.

Kaplan-Meier curves were used to estimate the incidence of first acute rejection, cumulative recipient survival, and cumulative incidence of graft loss, with between-group comparisons made using the log-rank test. The impact of different induction regimens on acute rejection was assessed using a multivariable Cox proportional hazards model, adjusting for confounding factors. P-values <0.05 were considered statistically significant.

### Propensity score matching (PSM)

This retrospective observational study included two non-randomized induction therapy groups (no induction and basiliximab [BSX] induction), potentially introducing confounding factors between them. For instance, the no induction group had fewer HLA mismatches and a shorter median dialysis duration, while recipients of extended criteria donor (ECD) kidneys were more likely to receive BSX induction. These differences could potentially compromise the reliability of the study conclusions.

To minimize bias arising from these imbalances, we employed propensity score matching (PSM) [23] to adjust for confounding factors. We calculated the standardized mean difference (SMD) for baseline characteristics, identifying variables with an SMD >0.1 as confounders to be included in the PSM analysis.

Propensity scores were estimated using a multivariable logistic regression model. A 2:1 nearest-neighbor matching without replacement was performed between the no induction and BSX induction groups, using a caliper width of 0.1 times the standard deviation of the logit of the propensity score.

Covariates used in the matching process included recipient body mass index (BMI), number of HLA mismatches, dialysis duration, and etiology of end-stage renal disease (ESRD). This process resulted in the matching of 35 recipients from the no induction group with 64 recipients from the BSX induction group.

After PSM, the distribution of confounding factors was well balanced between the two groups, with standardized mean difference (SMD) values less than 0.2 considered acceptable (Figure S2(A) and Table S1).

## Result

### Baseline characteristics

**Figure 1.**
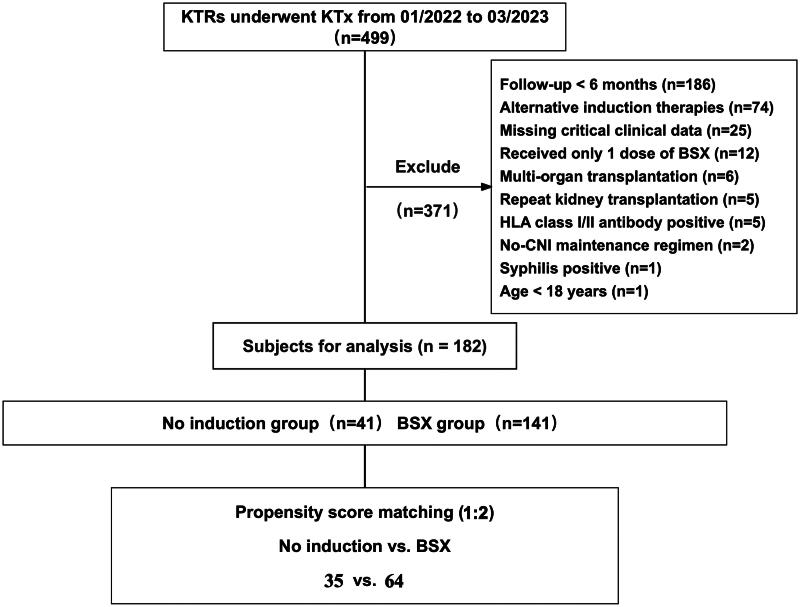
Flowchart of the study population. A total of 182 kidney transplant recipients (KTRs) were included in the study and divided into the no induction group and BSX group, with propensity score matching (PSM) performed. No induction: no biological agent induction; BSX: basiliximab induction.

Before PSM, baseline characteristics differed between the two groups (Table 1). The no induction group had significantly fewer HLA mismatches compared to the basiliximab (BSX) group (*p* < 0.001). Specifically, 85.7% of all kidney transplant recipients (KTRs) had ≤4 HLA mismatches before PSM, with 81.6% in the BSX group and 100% in the no induction group meeting this criterion. After PSM, 100% of recipients in both the BSX and no induction groups had ≤4 HLA mismatches (*p* = 0.737), while in the PSM-matched cohort, 89.1% of KTRs in the BSX group and 85.7% in the no induction group had ≤3 HLA mismatches. The main causes of end-stage renal disease (ESRD) were glomerulonephritis (17.0%) and hypertensive nephropathy (9.34%), with 60.4% of cases having an unknown etiology. Among the recipients, 28.6% received extended criteria donor (ECD) kidneys, 30.8% received kidneys from donors with acute kidney injury (AKI), and 83.0% underwent hemodialysis with a median duration of 24 months. All donors were donation after brain death (DBD). Exclusion criteria were: Follow-up less than 6 months (*n* = 186); Receiving other induction regimens (*n* = 74); Incomplete clinical data (*n* = 25); Incomplete BSX dosing (*n* = 12); Multi-organ transplantation (*n* = 6); Repeat kidney transplantation (*n* = 5); Positive preoperative HLA antibodies (*n* = 5); Maintenance regimens lacking calcineurin inhibitors (*n* = 2); Positive syphilis (*n* = 1); Age under 18 (*n* = 1) (Figure 1).

**Table 1.** Demographics and baseline characteristics of the study population.

	Total cohort (*n* = 182)	No induction (*n* = 41)	BSX (*n* = 141)	p*
Recipient gender, *n* (%)				0.896
Female	57 (31.3%)	12 (29.3%)	45 (31.9%)	
Male	125 (68.7%)	29 (70.7%)	96 (68.1%)	
Recipient age (yr), median [IQR]	36.5 [31.0;47.0]	37.0 [31.0;47.0]	36.0 [31.0;47.0]	0.977
Recipient BMI (kg/m^2^), Median [IQR]	22.0 [19.7;24.2]	21.3 [19.6;23.4]	22.1 [20.0;24.3]	0.161
HLA mismatch number, Median [IQR]	3.00 [3.00;4.00]	3.00 [2.00;3.00]	3.00 [3.00;4.00]	<0.001
Cause of ESRD, *n* (%)				0.052
Glomerulonephritis	31 (17.0%)	12 (29.3%)	19 (13.5%)	
Hypertensive nephropathy	17 (9.34%)	2 (4.88%)	15 (10.6%)	
Diabetic kidney disease	14 (7.69%)	0 (0.00%)	14 (9.93%)	
Polycystic kidney disease	2 (1.10%)	0 (0.00%)	2 (1.42%)	
Other	8 (4.40%)	2 (4.88%)	6 (4.26%)	
Unknown	110 (60.4%)	25 (61.0%)	85 (60.3%)	
Dialysis type, *n* (%)				0.578
Hemodialysis	151 (83.0%)	35 (85.4%)	116 (82.3%)	
Peritoneal dialysis	25 (13.7%)	6 (14.6%)	19 (13.5%)	
Preemptive transplantation	6 (3.30%)	0 (0.00%)	6 (4.26%)	
Dialysis duration (month), median [IQR]	23.0 [12.0;36.8]	24.0 [14.0;48.0]	22.0 [12.0;36.0]	0.328
Donor gender, *n* (%)				0.624
Female	33 (18.1%)	9 (22.0%)	24 (17.0%)	
Male	149 (81.9%)	32 (78.0%)	117 (83.0%)	
Donor age (yr), median [IQR]	48.0 [37.2;58.0]	48.0 [41.0;55.0]	48.0 [37.0;58.0]	0.841
Donor BMI (kg/m^2^), median [IQR]	23.5 [20.9;25.4]	24.0 [21.2;25.4]	23.0 [20.8;25.7]	0.934
Donor creatinine(μmol/L), Median [IQR]	99.0 [72.0;160]	89.0 [70.0;157]	100 [76.0;160]	0.357
AKI donor kidney, *n* (%)				0.668
AKI	56 (30.8%)	11 (26.8%)	45 (31.9%)	
No-AKI	126 (69.2%)	30 (73.2%)	96 (68.1%)	
Donor complication, *n* (%)				0.512
Diabetes	8 (4.40%)	3 (7.32%)	5 (3.55%)	
Hypertension	60 (33.0%)	14 (34.1%)	46 (32.6%)	
No	114 (62.6%)	24 (58.5%)	90 (63.8%)	
Donor type, *n* (%)				0.758
ECD	52 (28.6%)	13 (31.7%)	39 (27.7%)	
SCD	130 (71.4%)	28 (68.3%)	102 (72.3%)	

Data are expressed as mean ± standard deviation, number only, or number (%).

ECD: expanded criteria donor; SCD: standard criteria donor; AKI: acute kidney injury; HLA: human leukocyte antigen; preemptive transplantation, transplantation before dialysis; donor creatinine, serum creatinine value before transplantation; *Mann–Whitney *U* test and Chi-square test.

The median follow-up period was 12 months for both groups. Recipients in the no induction group received 1000 mg of methylprednisolone before graft reperfusion, tapered over three days postoperatively, with a median total dose of 35.1 mg/kg (30.8–39.2). All recipients in the BSX group received a total dose of 40 mg basiliximab and were treated with methylprednisolone prior to basiliximab infusion, which was gradually tapered within three days postoperatively. The median total dose of methylprednisolone administered to the BSX group was 32.5 mg/kg (28.6–37.7) (Table S2). After PSM adjustment, baseline covariates were well balanced, with no significant differences remaining between the groups (Table S3).

### Primary indicators

At 12 months post-transplantation, 28 kidney transplant recipients (KTRs) experienced their first acute rejection (AR) episode (Table 2), including 5 cases in the no induction group and 23 in the BSX group. Of these, 20 cases were diagnosed based on clinical criteria.

**Table 2.** Summary of acute rejection outcomes.

	Total cohort (*n* = 182)	No induction (*n* = 41)	BSX (*n* = 141)	p*
AR, *n* (%)	28 (15.4%)	5 (12.2%)	23 (16.3%)	0.619
BPAR	8 (4.40%)	0 (0.00%)	8 (5.67%)	0.202
TCMR_IB	2 (1.10%)	0 (0.00%)	2 (1.42%)	1.000
TCMR_IIA	2 (1.10%)	0 (0.00%)	2 (1.42%)	1.000
Borderline change	4 (2.20%)	0 (0.00%)	4 (2.84%)	0.576
Clinically diagnosed	20 (11.0%)	5 (12.2%)	15 (10.6%)	0.779
Steroid resistant	5 (2.75%)	1 (2.44%)	4 (2.84%)	1.000
AR-time(day), median [IQR]	18.0 [11.0;75.0]	11.0 [9.0;18.0]	19.0 [11.0;75.0]	0.301
Treatment, *n* (%)				
MP	22 (12.1%)	4 (9.76%)	18 (12.8%)	0.787
r-ATG	1 (0.55%)	0 (0.00%)	1 (0.71%)	1.000
MP + r-ATG	1 (0.55%)	0 (0.00%)	1 (0.71%)	1.000
MP + plasmapheresis	5 (2.75%)	0 (0.00%)	1 (0.71%)	1.000

AR: acute rejection; BPAR: biopsy-confirmed acute rejection; TCMR: T-cell mediated rejection; ABMR: antibody-mediated rejection; MP: methylprednisolone; r-ATG: rabbit anti-human T-lymphocyte porcine immunoglobulin; AR-time: time to first acute rejection; *Mann–Whitney *U* test and Chi-square test.

According to the Banff 2019 classification, 8 cases were biopsy-proven acute rejection (BPAR): 2 cases of T cell-mediated rejection (TCMR) grade IIA, 2 cases of TCMR grade IB, and 4 cases of borderline changes. Both TCMR grade IIA cases occurred in the BSX group; no grade II or higher rejections were observed in the no induction group. There was no significant difference in the incidence or severity of BPAR between the two groups (*p* = 0.202). All 5 AR cases in the no induction group were clinically diagnosed, with 4 responding to corticosteroid treatment. Steroid-resistant AR was rare, with 4 cases occurring in the BSX group.

The median time to first AR was 11 days in the no induction group and 19 days in the BSX group (*p* = 0.301), showing no significant difference. The cumulative incidence of first AR did not differ significantly between the groups (log-rank *p* = 0.46, Figure S2(B) and Table S4).

**Figure 2.**
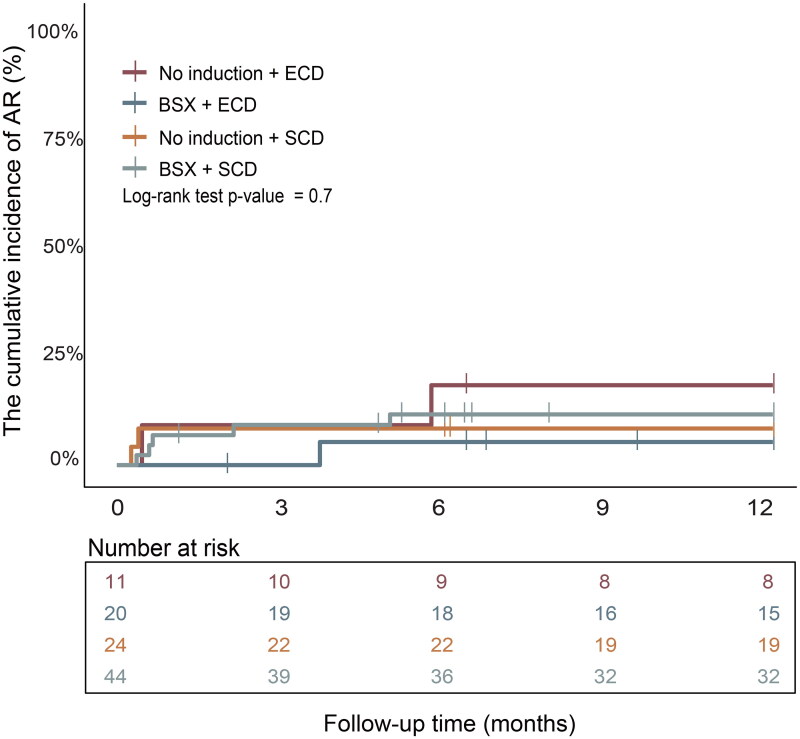
The incidence of acute rejection (AR) among groups. The cumulative incidence of AR after receiving an ECD or SCD transplant in recipients treated with no induction or BSX groups.

After PSM, subgroup analyses based on induction regimens and donor types (ECD and SCD) revealed no significant differences in cumulative AR incidence between the groups (Figure 2).

## Secondary indicators

### Recipient death and graft loss

Over the 12-month follow-up period, 20 recipients (8.85%) died, with severe pneumonia being the leading cause of death (14 cases). Mortality was lower in the no induction group (1 case) compared to the basiliximab (BSX) group (15 cases) (Table S5). However, Kaplan–Meier analysis showed no significant difference in cumulative survival rates between the groups (log-rank *p* = 0.11, Figure S2(C)).

Excluding deaths, 7 recipients (3.1%) experienced graft loss. Causes included antibody-mediated rejection (ABMR) (1 case), T cell-mediated rejection (TCMR) (1 case), focal segmental glomerulosclerosis (FSGS) (1 case), and unidentified causes (1 case). No graft dysfunction occurred in the no induction group, while 4 cases (2.84%) were observed in the BSX group (Table S5). The cumulative incidence of graft loss at 12 months showed no significant difference between the groups (log-rank *p* = 0.27, Figure S2(D)).

### Delayed graft function (DGF)

During follow-up, 33 recipients (18.1%) experienced DGF, with a lower incidence in the no induction group (9.76%, 4/41) compared to the BSX group (20.6%, 29/141), although the difference did not reach statistical significance (*p* = 0.177, Table S6). Creatinine levels (maximum and minimum) during the DGF period were comparable between the groups, with no statistically significant differences observed.

### Renal graft function

**Figure 3.**
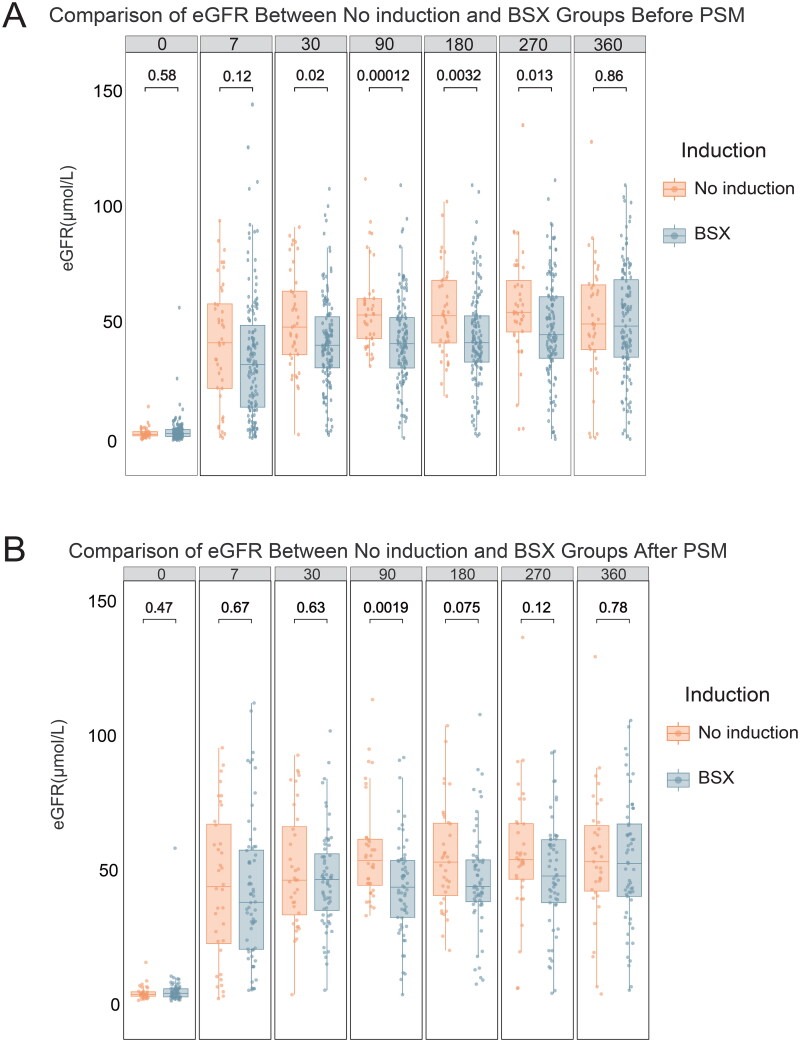
(A) The overall eGFR between the two groups within 12 months before PSM; (B) Comparison of eGFR between the no induction and BSX groups after PSM.

The median estimated glomerular filtration rate (eGFR) at 30-, 90-, 180-, and 270-days post-transplantation was significantly higher in the no induction group compared to the BSX group. At 270 days, the eGFR in the no induction group was 57.0 mL/min/1.73 m^2^, significantly higher than 49.0 mL/min/1.73 m^2^ in the BSX group (*p* = 0.013, Figure 3(A)). Even after PSM and pairwise comparisons, the eGFR at multiple time points post-transplantation in the no induction group showed comparable outcomes to the BSX group, with a significant difference at 90 days (*p* = 0.0019, Figure 3(B)).

### Adverse events

There was a significant difference in the overall incidence of adverse events between the two groups. The overall incidence of adverse events in the no induction group was 29.3%, significantly lower than 48.2% in the BSX group (*p* = 0.048). Among these, infections requiring hospitalization were the most common adverse events. The total incidence of infections in the no induction group was 29.3%, lower than 40.4% in the BSX group, although the difference was not statistically significant (*p* = 0.266). However, the incidence of respiratory infections in the BSX group was significantly higher than that in the no induction group (*p* = 0.008). There were no cases of thrombocytopenia in the no induction group, while the incidence in the BSX group was 12.8% (*p* = 0.014). The incidence of leukopenia showed no statistically significant difference between the two groups (*p* = 0.589) (Table 3).

**Table 3.** Adverse events occurring within 12 months post-transplantation.

	Total cohort (*n* = 182)	No induction (*n* = 41)	BSX (*n* = 141)	p*
Adverse events	80 (44.0%)	12 (29.3%)	68 (48.2%)	0.048
Infections requiring- hospitalization	69 (37.9%)	12 (29.3%)	57 (40.4%)	0.266
Urinary	19 (10.4%)	5 (12.2%)	14 (9.93%)	0.772
Respiratory	44 (24.2%)	3 (7.32%)	41 (29.1%)	0.008
Bloodstream	13 (7.14%)	3 (7.32%)	10 (7.09%)	1.000
Surgical site	2 (1.10%)	0 (0.00%)	2 (1.42%)	1.000
G+	22 (12.1%)	2 (4.88%)	20 (14.2%)	0.171
G-	13 (7.14%)	4 (9.76%)	9 (6.38%)	0.493
Fungal	22 (12.1%)	2 (4.88%)	20 (14.2%)	0.171
Viral	50 (27.5%)	8 (19.5%)	42 (29.8%)	0.272
Pneumocystis	9 (4.95%)	1 (2.44%)	8 (5.67%)	0.686
Legionella	2 (0.88%)	1 (2.44%)	1 (0.71%)	0.368
CMV	11 (6.04%)	2 (4.88%)	9 (6.38%)	1.000
BK Viruria	16 (8.79%)	3 (7.32%)	13 (9.22%)	0.401
BK Viremia	5 (2.75%)	1 (2.44%)	4 (2.84%)	1.000
Leukopenia	5 (2.75%)	0 (0.00%)	5 (3.55%)	0.589
Thrombocytopenia	18 (9.89%)	0 (0.00%)	18 (12.8%)	0.014

G+: Gram-positive bacteria; G−: Gram-negative bacteria; BK Viruria: BKPyV DNA > 10^7^copies/ml; BK Viremia: BKPyV DNA > 10^4^ copies/ml; *Mann–Whitney *U* test and Chi-square test.

### Maintenance immunosuppression regimen

All kidney transplant recipients (KTRs) were discharged with the standard triple therapy consisting of tacrolimus (TAC), mycophenolic acid (MPA), and prednisone (Pred). Over time, the proportion of recipients maintaining the standard triple therapy gradually declined in both groups. At 12 months post-transplantation, 78.0% of the recipients in the no induction group still received the standard triple therapy, which was higher than 62.4% in the BSX group (*p* = 0.094, Figure S3(A)).

### Tacrolimus (TAC) trough level

The trough levels of tacrolimus in both groups remained within the target range during the 12-month follow-up period. At 7 days post-transplantation, the trough concentration of tacrolimus in the no induction group was significantly lower than that in the BSX group (*p* = 0.04). There were no significant differences between the two groups at the remaining time points post-transplantation (Figure S3(B)).

### Multivariate Cox regression analysis

Table S7 summarizes the outcomes of the multivariable Cox regression analyses conducted on the four models after PSM. Model 1 did not include any covariates. BSX induction did not significantly reduce the risk of acute rejection compared to no induction (BSX group hazard ratio [HR] = 1.81, 95% confidence interval [CI], 0.23–2.88, *p* = 0.748). Model 2 incorporated donor-related variables and showed similar results to Model 1 (BSX group HR = 1.02, 95% CI, 0.25–4.08, *p* = 0.982). Model 3 included recipient-related variables, and the HR decreased, but the difference was not statistically significant (BSX group HR = 0.57, 95% CI, 0.16–2.07, *p* = 0.4). Model 4 combined donor- and recipient-related variables, reducing the HR for the BSX group to 1.35 (95% CI, 0.37–4.88, *p* = 0.650); however, the difference remained nonsignificant. By continuously including different covariates for comparison, BSX failed to significantly reduce the risk of first acute rejection compared to no induction in any of the different models.

## Discussion

In 2009, the Kidney Disease: Improving Global Outcomes (KDIGO) guidelines provided recommendations for induction therapy protocols, suggesting that recipients with low-immunological-risk conventionally receive non-lymphocyte-­depleting antibodies such as interleukin-2 receptor antagonists (IL-2RA) for induction. In 2010, a Cochrane review synthesized the clinical trials encompassed in this guideline [24], revealing that the maintenance immunosuppressive regimens in numerous studies were primarily based on cyclosporine A (CsA) and azathioprine (AZA). Specifically, 87% of kidney transplant recipients (KTRs) received CsA, 28% received AZA, and a larger proportion of recipients were administered dual regimens rather than triple regimens. After conducting a meta-analysis of 24 clinical studies, it was determined that regardless of the immunological risk level, the administration of IL-2RA did not significantly influence all-cause mortality or renal allograft dysfunction. However, high-risk recipients could benefit from IL-2RA in terms of acute rejection prevention (*p* = 0.02). According to the Organ Procurement and Transplantation Network (OPTN) report [5], in 2022, 92.1% of adult KTRs in the United States underwent biological agent induction during surgery, with basiliximab (BSX) being the most prevalently used regimen. The situation is similar in China. However, considering Guangxi Zhuang Autonomous Region, a less developed region with a considerable number of low-income individuals, not all recipients can afford the high cost of induction regimens. Induction therapy also increases the risk of early postoperative infections, particularly for recipients in Guangxi, which prolongs the length of stay in the intensive care unit (ICU) and extends the duration of preventive anti-infection treatment, thereby increasing hospitalization expenses. For this population, conducting thorough immunological risk assessment, weighing the pros and cons, and designing individualized induction protocols is of utmost importance.

We conducted a single-center retrospective cohort study, comprehensively comparing the safety and efficacy of no induction and BSX induction among low-immunological-risk recipients. During the entire follow-up period, the cumulative incidence of the first acute rejection episode at 12 months was similar in the two groups. The outcomes demonstrated no significant difference even after stratifying based on ECD and SCD in the cohort subsequent to PSM. We also established four multivariable Cox proportional hazards models within the cohort after PSM. By adjusting for various covariates, there was no statistical difference in the risk of acute rejection between the two groups, although the hazard ratio (HR) values fluctuated. The effectiveness of no induction and BSX in preventing acute rejection was comparable. There were no significant differences in DGF, all-cause mortality, or graft loss between the two groups. Considering the relatively small number of events following PSM, no comparisons were carried out for the aforementioned outcomes after PSM.

Currently, numerous studies have revealed that BSX induction only exhibits a distinct advantage in recipients undergoing an immunosuppressive regimen predominantly constituted by CsA [25,26]. However, for those receiving a regimen primarily based on tacrolimus (TAC), the effect is insignificant, according to short-term and long-term follow-up. Lim et al. retrospectively analyzed 1,220 low-risk recipients and, when conducting subgroup analysis based on different calcineurin inhibitor (CNI) regimens, discovered that the administration of IL-2RA reduced the risk of ARE in recipients receiving CsA (*p* < 0.001) but had no discernible improvement effect in recipients receiving TAC (*p* = 0.48) [27]. However, only 218 recipients received IL-2RA, and no reports were provided on the induction regimens for the remaining 1,008 recipients. They were simply combined, with an excessive number of potential confounding factors among the groups. Tanriover et al. included KTRs who received living donor kidney transplants from 2000 to 2012 and were discharged with TAC/MPA and steroid maintenance. In the group with long-term steroid maintenance, compared with IL-2RA, no induction did not increase the risk of acute rejection at 1 year, and the 5-year cumulative survival rates of recipients among the three groups were comparable. Induction therapy did not lead to better prognostic outcomes. However, in the population who discontinued steroids prematurely, IL-2RA and rabbit antithymocyte globulin (r-ATG) induction was a significant option [28]. Evans et al. retrospectively analyzed low-risk KTRs with well-matched human leukocyte antigen (HLA)-A, -B, -DR, and -DQ loci between donors and recipients from 2010 to 2014 and followed up until 2017. The study found that for different observed outcomes (including graft loss after transplantation due to any cause or acute rejection, with or without the inclusion of death), there were no significant differences between the two groups [29]. After further stratification based on calculated panel reactive antibody (cPRA), in the subpopulations with cPRA of 0, 1–29, 30–79, 80–97, and 98–100%, there was no significant difference in the cumulative incidence of graft loss (from any cause) between the IL-2RA/no induction group and the T-cell depletion antibody induction group. Perhaps for well-matched low-risk KTRs, no form of induction might be necessary. In recent years, several meta-analyses have compared the results of different induction regimens in low-risk recipients, indicating that compared with no induction, IL-2RA induction does not reduce the risk of acute rejection or improve recipient and graft outcomes [10,30,31].

Consistent with our research findings, the incidence of postoperative infections requiring hospitalization was significantly lower in the no induction group than in the BSX group, particularly for respiratory infections. This reduction could shorten ICU stays, decrease the duration of prophylactic anti-infective treatments, and lower overall hospitalization costs, reducing the consumption of healthcare resources. Furthermore, recipients undergoing BSX induction were more prone to hematological system impairments such as thrombocytopenia, which is indicative of excessive short-term immune suppression, increasing the risks of infection, early graft dysfunction, and recipient mortality.

Additionally, the changes in the triple maintenance immunosuppressive regimen were relatively stable in the no induction group. The proportion of recipients on TAC/MPA/Pred in the no induction group at 12 months was higher than that in the BSX group, and the TAC trough levels in both groups were within the target range. Even in the early postoperative period, the no induction group did not require high TAC trough levels, which supported the recovery of short-term graft function. The stability of the standard triple maintenance regimen in the no induction group within 12 months reinforced recipients’ confidence in long-term follow-up.

The eGFR values of recipients in the no induction group surpassed those in the BSX group at multiple time points within 12 months post-transplantation. This advantage emerged starting from 30 days post-transplantation and persisted until 270 days. Even after further PSM, this trend persisted between the two groups. The recovery of graft function in the no induction group was significantly faster. However, with prolonged observation, this gap gradually narrowed, and by 12 months, there was no significant difference in eGFR values between the two groups. This is similar to the results of a retrospective study conducted in the United Kingdom in 2020 [25]. At 3 months, the eGFR of recipients in the no induction group was significantly higher than that in the induction group (*p* < 0.0001), but there was no significant difference in eGFR values between the two groups at 1 year.

Currently, there are no large-scale, multicenter, blinded, prospective, randomized controlled trials (RCTs) comparing induction regimens for low-risk recipients who solely employ TAC-based immunosuppressive maintenance regimens. The estimated sample size capable of demonstrating differences in the clinical outcomes of different induction regimens ranges between 1600 and 7000 [13]. Such research incurs extremely high costs, and the extensive use of induction regimens required for the study also brings numerous unpredictable risks. The optimal induction regimen for low-immunological-risk recipients still requires verification through large-scale RCTs. The majority of existing studies are retrospective analyses based on large databases. These data inherently possess selection biases, the backgrounds of the included recipients are heterogeneous, and the differences between groups are substantial. The definitions of low immunological risk remain controversial, and different researchers have their own standards. Laftavi et al. suggested that race is a risk factor [32]; Tanriover et al. considered recipients of living donor kidneys to have lower immunological risk [28]; Lee et al. classified recipients with preoperative PRA <30% as low-risk [33]; Jha et al. defined low immunological risk by excluding high-risk factors [34]; and other researchers have suggested that older recipients are low-risk [35]. Furthermore, many studies lack laboratory indicators of interest, such as eGFR and TAC trough levels, and the rationale for selecting induction regimens is often not clearly explained. Commonly, the basis for selecting the induction regimen is not explained. A considerable number of studies still incorporate some maintenance regimens mainly based on CsA, and very few studies report the changes in maintenance immunosuppressive regimens and TAC trough levels. The definitions of acute rejection episodes are also not uniform.

The population included in our study all received the standard triple maintenance immunosuppressive regimen (TAC/MPA/Pred) at discharge. All recipients were undergoing their first kidney transplantation, were preoperatively PRA-negative, had negative HLA class I and II antibodies, and ABO blood type compatibility. All recipients received kidneys from donors after brain death (DBD), with a cold ischemia time (CIT) of less than 12 h. Additionally, 85.7% of kidney transplant recipients (KTRs) had ≤4 HLA mismatches, indicating an extremely low immunological risk. They had the same long-term maintenance regimen, regular outpatient follow-ups post-transplantation, and we reported the changes in the maintenance immunosuppressive regimen and TAC trough levels within 12 months. The definition of BPAR was based on the 2009 Banff classification, while PSM was simultaneously applied to adjust for confounding factors between groups, ensuring the reliability of the results.

Advances in immunology and genetics have revolutionized the assessment criteria for immunological risk in the past. Previously, we evaluated the immunological risk of recipients via panel reactive antibody (PRA). However, with the advent of single-antigen bead testing [36], laboratories have acquired the capacity to detect and identify HLA antibodies with extremely high sensitivity and specificity. These developments prompted the United Network for Organ Sharing (UNOS) to initiate the utilization of calculated panel reactive antibody (cPRA) for assessing the sensitization degree of recipients in 2009 [37]. OPTN defines high sensitization as cPRA ranging from 98 to 100%. Clinical prediction models constructed using machine learning methods differ from traditional diagnostic approaches, enhancing predictive and classification capabilities [38]. Based on the enhancement of the aforementioned assessment methods, recipients who were previously considered high-risk might be classified as having low immunological risk under the new standards. How to design the optimal individualized induction protocols for these recipients still demands more research for verification.

There are certain limitations to our study. It is a single-center retrospective cohort investigation, and some recipients with low immunological risk were administered no induction treatment for a variety of reasons. We retrospectively reviewed the outcomes of this group of individuals. In our center, where access to graft biopsy is limited, many cases of acute rejection were diagnosed clinically. Although we strictly established clinical diagnostic criteria in our center, this undoubtedly weakens the reliability of the results to some extent. Simplifying and accelerating the diagnostic process will be necessary in the future. This approach can also be further extended to the evaluation of living donor kidney transplantation and pediatric donor kidney transplantation. Considering the potential risks associated with no induction treatment, expanding the sample size to a sufficient magnitude proved challenging. Geographical and demographic factors may limit the generalizability of the study results, but our research included recipients from various regions across China, such as Guangxi, Heilongjiang, Beijing, Shanghai, and Hangzhou. The study population was not limited to the Han ethnicity but also included ethnic minorities like the Zhuang and Yao. As a result, our findings may provide insights into the broader transplant landscape in China. Future validation is required through national multi-center studies. In Southeast Asia, in many member countries of the Association of Southeast Asian Nations (ASEAN), where economic development and national policies result in a reliance on living donor kidney transplants, recipients of such transplants are generally considered to have low immunological risk. Our findings could serve as a valuable reference for these populations. Researchers in the United Kingdom, the United States, and Australia have also drawn similar conclusions through studies based on large databases. In conclusion, our study discovered that in recipients with low immunological risk, the no induction treatment protocol might be a safe and effective option.

## Supplementary Material

Figure S1.pdf

Table.docx

Figure legends.docx

Graphical abstract.pdf

Figure S3.pdf

Figure 1.pdf

Supplement Material.docx

Figure 2.pdf

Figure S2.pdf

Figure 3.pdf
