# Supplementary material for: Evaluating the efficacy of basiliximab versus no induction in low-immunological-risk kidney transplant recipients: a propensity score matched analysis
Source: Ren Fail. 2025 Feb 20;47(1):2460729. doi: 10.1080/0886022X.2025.2460729 (PMC11843659; doi:10.1080/0886022X.2025.2460729)
Supplement: Figure S1.pdf [file IRNF_A_2460729_SM4961.pdf]

# Figure S1

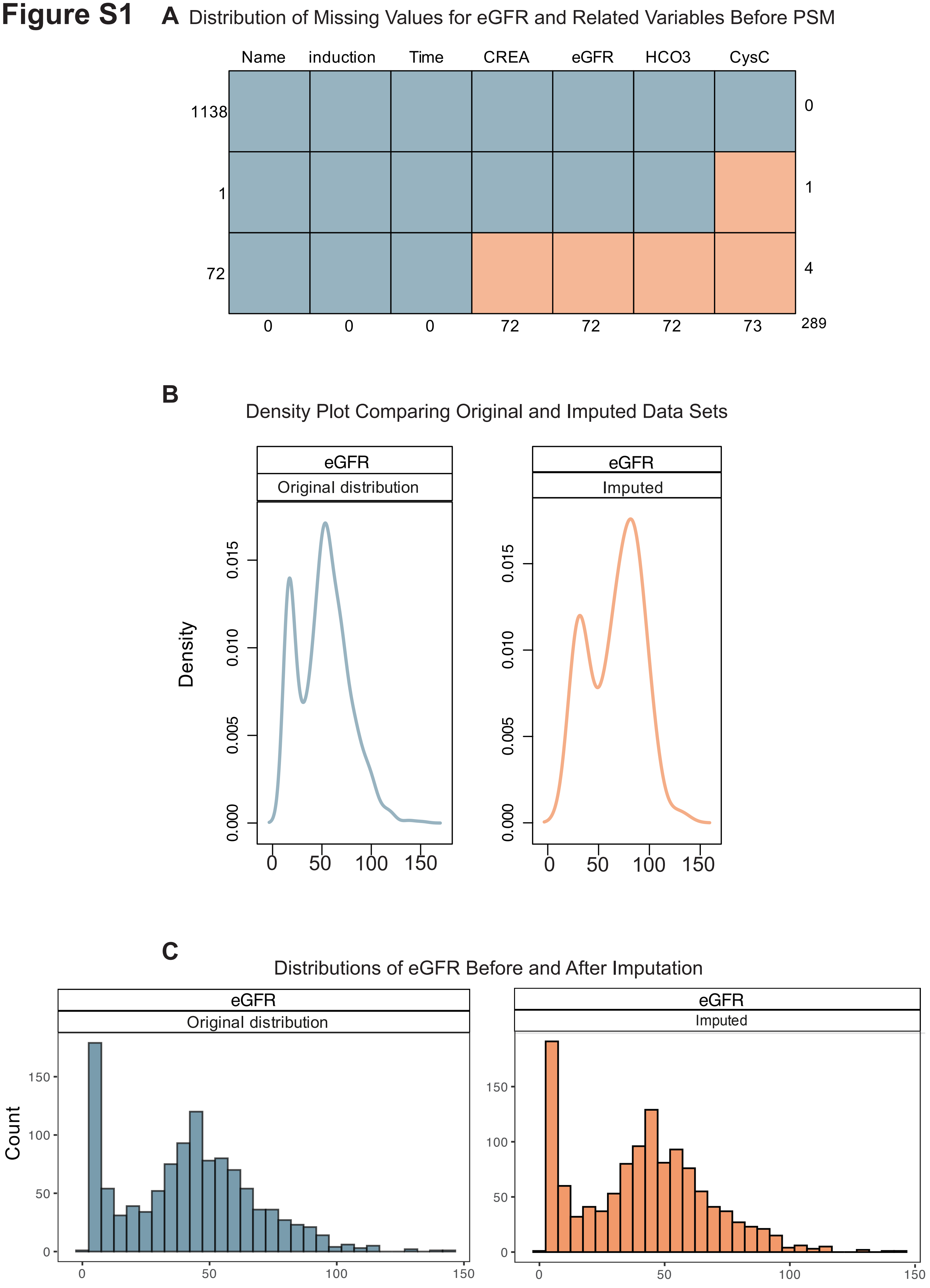
A Distribution of Missing Values for eGFR and Related Variables Before PSM
Name
induction
Time
CREA
eGFR
HCO3
CysC
1138
1
72
0
1
4
0
0
0
72
72
72
73
289
B
Density Plot Comparing Original and Imputed Data Sets
eGFR
Original distribution
eGFR
Imputed
Density
0.000
0.005
0.010
0.015
0
50
100
150
C
Distributions of eGFR Before and After Imputation
eGFR
Original distribution
eGFR
Imputed
Count
0
50
100
150
