# Supplementary material for: Evaluating the efficacy of basiliximab versus no induction in low-immunological-risk kidney transplant recipients: a propensity score matched analysis
Source: Ren Fail. 2025 Feb 20;47(1):2460729. doi: 10.1080/0886022X.2025.2460729 (PMC11843659; doi:10.1080/0886022X.2025.2460729)
Supplement: Table.docx [file IRNF_A_2460729_SM4960.docx]

Table 1. Demographics and baseline characteristics of the study population

|  | | Total cohort （n=182） | No Induction  （n=41） | BSX  （n=141） | p* |
| --- | --- | --- | --- | --- | --- |
| Recipient Gender, n (%) | |  |  |  | 0.896 |
| female | | 57 (31.3%) | 12(29.3%) | 45 (31.9%) |  |
| male | | 125 (68.7%) | 29 (70.7%) | 96 (68.1%) |  |
| Recipient Age(yr), Median [IQR] | | 36.5 [31.0;47.0] | 37.0 [31.0;47.0] | 36.0 [31.0;47.0] | 0.977 |
| Recipient BMI (kg/m^2^), Median [IQR] | | 22.0 [19.7;24.2] | 21.3 [19.6;23.4] | 22.1 [20.0;24.3] | 0.161 |
| HLA mismatch number, Median [IQR] | | 3.00[3.00;4.00] | 3.00[2.00;3.00] | 3.00[3.00;4.00] | <0.001 |
| Cause of ESRD, n (%) | |  |  |  | 0.052 |
| Glomerulonephritis | | 31 (17.0%) | 12 (29.3%) | 19 (13.5%) |  |
| Hypertensive nephropathy | | 17 (9.34%) | 2(4.88%) | 15 (10.6%) |  |
| Diabetic kidney disease | | 14 (7.69%) | 0(0.00%) | 14 (9.93%) |  |
| Polycystic kidney disease | | 2 (1.10%) | 0(0.00%) | 2(1.42%) |  |
| Other | | 8 (4.40%) | 2(4.88%) | 6 (4.26%) |  |
| Unknown | | 110 (60.4%) | 25(61.0%) | 85(60.3%) |  |
| Dialysis type, n (%) | |  |  |  | 0.578 |
| Hemodialysis | | 151 (83.0%) | 35(85.4%) | 116(82.3%) |  |
| Peritoneal dialysis | | 25 (13.7%) | 6 (14.6%) | 19(13.5%) |  |
| Preemptivetransplantation | | 6 (3.30%) | 0(0.00%) | 6 (4.26%) |  |
| Dialysis duration (month), Median [IQR] | | 23.0 [12.0;36.8] | 24.0[14.0;48.0] | 22.0 [12.0;36.0] | 0.328 |
| Donor Gender, n (%) | |  |  |  | 0.624 |
| female | | 33 (18.1%) | 9(22.0%) | 24 (17.0%) |  |
| male | | 149 (81.9%) | 32(78.0%) | 117(83.0%) |  |
| Donor Age (yr), Median [IQR] | | 48.0 [37.2;58.0] | 48.0 [41.0;55.0] | 48.0 [37.0;58.0] | 0.841 |
| Donor BMI (kg/m^2^), Median [IQR] | | 23.5 [20.9;25.4] | 24.0[21.2;25.4] | 23.0 [20.8;25.7] | 0.934 |
| Donor creatinine(μmol/L), Median [IQR] | | 99.0 [72.0;160] | 89.0[70.0;157] | 100[76.0;160] | 0.357 |
| AKI donor kidney, n (%) | |  |  |  | 0.668 |
| AKI | | 56 (30.8%) | 11(26.8%) | 45(31.9%) |  |
| No-AKI | | 126 (69.2%) | 30(73.2%) | 96(68.1%) |  |
| Donor Complication, n (%) | |  |  |  | 0.512 |
| Diabetes | | 8 (4.40%) | 3 (7.32%) | 5 (3.55%) |  |
| Hypertension | | 60 (33.0%) | 14 (34.1%) | 46 (32.6%) |  |
| No | | 114 (62.6%) | 24 (58.5%) | 90 (63.8%) |  |
| Donnor type, n (%) | |  |  |  | 0.758 |
| ECD | | 52 (28.6%) | 13(31.7%) | 39(27.7%) |  |
| SCD | | 130 (71.4%) | 28(68.3%) | 102(72.3%) |  |
| Data are expressed as mean ± standard deviation, number only, or number (%) ECD, Expanded Criteria Donor; SCD, Standard Criteria Donor; AKI, Acute Kidney Injury; HLA, Human Leukocyte Antigen; Preemptive transplantation, Transplantation before dialysis; Donor creatinine, Serum creatinine value before transplantation; *Mann-Whitney U test and Chi-square test | | | | | |
|  |  |  |  |  |  |

Table 2. Summary of Acute Rejection Outcomes

|  | Total cohort （n=182） | | No Induction （n=41） | | BSX （n=141） | | P* |
| --- | --- | --- | --- | --- | --- | --- | --- |
| AR, n (%) | 28 (15.4%) | | 5(12.2%) | | 23(16.3%) | | 0.619 |
| BPAR | 8 (4.40%) | | 0(0.00%) | | 8(5.67%) | | 0.202 |
| TCMR_IB | 2 (1.10%) | | 0(0.00%) | | 2(1.42%) | | 1.000 |
| TCMR_IIA | 2 (1.10%) | | 0(0.00%) | | 2(1.42%) | | 1.000 |
| Borderline change | 4 (2.20%) | | 0(0.00%) | | 4(2.84%) | | 0.576 |
| Clinically diagnosed | 20 (11.0%) | | 5(12.2%) | | 15(10.6%) | | 0.779 |
| Steroid resistant | 5 (2.75%) | | 1(2.44%) | | 4(2.84%) | | 1.000 |
| AR-Time(day), Median [IQR] | 18.0[11.0;75.0] | | 11.0[9.0;18.0] | | 19.0[11.0;75.0] | | 0.301 |
| Treatment, n (%) |  | |  | |  | |  |
| MP | 22 (12.1%) | | 4(9.76%) | | 18(12.8%) | | 0.787 |
| r-ATG | 1 (0.55%) | | 0(0.00%) | | 1(0.71%) | | 1.000 |
| MP + r-ATG | 1 (0.55%) | | 0(0.00%) | | 1(0.71%) | | 1.000 |
| MP + plasmapheresis | 5 (2.75%) | | 0(0.00%) | | 1(0.71%) | | 1.000 |
| AR, Acute rejection; BPAR, Biopsy-confirmed acute rejection; TCMR, T-cell mediated rejection; ABMR：antibody-mediated rejection; MP, Methylprednisolone；r-ATG, rabbit anti-human T-lymphocyte porcine immunoglobulin；AR-Time, Time to first acute rejection;  *Mann-Whitney U test and Chi-square test | | | | | | | |
|  |  |  | |  | |  | |

Table 3. Adverse Events Occurring Within 12 Months Post-Transplantation.

|  | Total cohort  (n=182) | No induction  (n=41) | BSX (n=141) | P* |
| --- | --- | --- | --- | --- |
| Adverse events | 80 (44.0%) | 12 (29.3%) | 68 (48.2%) | 0.048 |
| Infections requiring- hospitalization | 69 (37.9%) | 12 (29.3%) | 57 (40.4%) | 0.266 |
| Urinary | 19 (10.4%) | 5 (12.2%) | 14 (9.93%) | 0.772 |
| Respiratory | 44 (24.2%) | 3 (7.32%) | 41 (29.1%) | 0.008 |
| Bloodstream | 13 (7.14%) | 3 (7.32%) | 10 (7.09%) | 1.000 |
| Surgical site | 2 (1.10%) | 0 (0.00%) | 2 (1.42%) | 1.000 |
| G+ | 22 (12.1%) | 2 (4.88%) | 20 (14.2%) | 0.171 |
| G- | 13 (7.14%) | 4 (9.76%) | 9 (6.38%) | 0.493 |
| Fungal | 22 (12.1%) | 2 (4.88%) | 20 (14.2%) | 0.171 |
| Viral | 50 (27.5%) | 8 (19.5%) | 42 (29.8%) | 0.272 |
| Pneumocystis | 9 (4.95%) | 1 (2.44%) | 8 (5.67%) | 0.686 |
| Legionella | 2 (0.88%) | 1 (2.44%) | 1 (0.71%) | 0.368 |
| CMV | 11 (6.04%) | 2 (4.88%) | 9 (6.38%) | 1.000 |
| BK Viruria | 16 (8.79%) | 3 (7.32%) | 13 (9.22%) | 0.401 |
| BK Viremia | 5 (2.75%) | 1 (2.44%) | 4 (2.84%) | 1.000 |
| Leukopenia | 5 (2.75%) | 0 (0.00%) | 5 (3.55%) | 0.589 |
| Thrombocytopenia | 18 (9.89%) | 0 (0.00%) | 18 (12.8%) | 0.014 |
| G+, Gram-positive bacteria; G-, Gram-negative bacteria; BK Viruria, BKPyV DNA＞10^7^copies/ml;  BK Viremia, BKPyV DNA＞10^4^ copies/ml;  *Mann-Whitney U test and Chi-square test | | | | |
