# Supplementary material for: Evaluating the efficacy of basiliximab versus no induction in low-immunological-risk kidney transplant recipients: a propensity score matched analysis
Source: Ren Fail. 2025 Feb 20;47(1):2460729. doi: 10.1080/0886022X.2025.2460729 (PMC11843659; doi:10.1080/0886022X.2025.2460729)
Supplement: Figure legends.docx [file IRNF_A_2460729_SM4958.docx]

**Figure 1. Flowchart of the study population.** A total of 182 kidney transplant recipients (KTRs) were included in the study and divided into the no induction group and BSX group , with propensity score matching (PSM) performed. **no induction**, no biological agent induction; **BSX**, Basiliximab induction.

**Figure 2. The incidence of acute rejection (AR) among groups.** The cumulative incidence of AR after receiving an ECD or SCD transplant in recipients treated with no induction or BSX groups.

**Figure 3**. (A)The overall eGFR between the two groups within 12 months before PSM; **(B)** Comparison of eGFR between the no induction and BSX groups after PSM.

**Figure S1**.(A) Distribution of Missing Values for eGFR and Related Variables Before PSM: The heatmap displays the missing data pattern for eGFR and its related variables (CREA, HCO3, and CysC). Each row represents a case, and each column represents a variable. Missing values are highlighted in orange.(B) Density Plot Comparing Original and Imputed Data Sets: The density plots illustrate the distribution of eGFR values before imputation ("Original distribution") and after imputation ("Imputed"). The imputed dataset shows a smoother and more complete distribution.(C) Distributions of eGFR Before and After Imputation: The histograms compare the distributions of eGFR values in the original dataset and the imputed dataset.

**Figure S2.** (A) A total of 64 kidney transplant recipients (KTRs) in the no induction group were matched with 35 KTRs in the BSX group. (B) Cumulative incidence of acute rejection among two induction protocols, no induction and BSX groups; (C) Cumulative incidence of graft loss within 12 months; (D). Cumulative survival within 12 months；

**Figure S3**. (A) Comparison of maintenance immunosuppression regimens among no induction and BSX groups Before PSM; (B) Comparison of TAC dose (ng/ml) among the two groups within 12 months Before PSM.
