# Supplementary material for: Evaluating the efficacy of basiliximab versus no induction in low-immunological-risk kidney transplant recipients: a propensity score matched analysis
Source: Ren Fail. 2025 Feb 20;47(1):2460729. doi: 10.1080/0886022X.2025.2460729 (PMC11843659; doi:10.1080/0886022X.2025.2460729)
Supplement: Graphical abstract.pdf [file IRNF_A_2460729_SM4956.pdf]

Evaluating the Efficacy of Basiliximab Versus No Induction in Low-Immunological-Risk Kidney Transplant Recipients: A Propensity Score Matched Analysis

Background

In low-immunological-risk kidney transplant recipients (KTRs), the role of induction therapy remains controversial

2022.01–2023.03
182 low-immunological-risk KTRs

After PSM : 99 KTRs
No induction group : 35 KTRs
BSX group : 64 KTRs

The First Acute Rejection, All-cause mortality Graft Loss, DGF, Renal Graft Function, Maintenance Immunosuppression and Infections

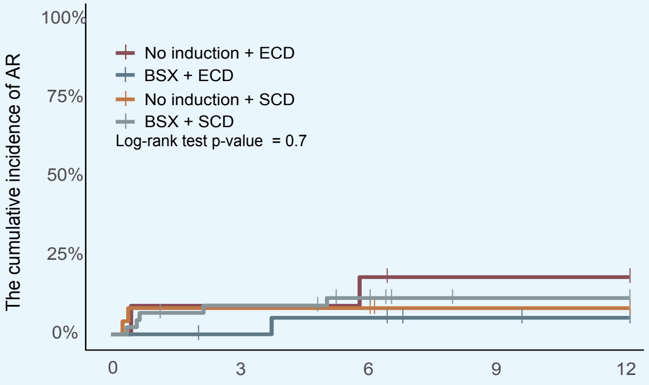

- After PSM, no induction therapy showed similar cumulative AR incidence between groups in subgroup analyses based on induction regimens and donor types

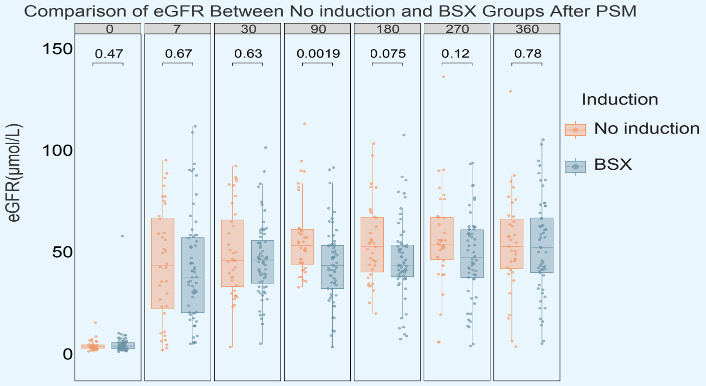

- After PSM, the no induction group showed similar eGFR at multiple early postoperative time points compared to the BSX group

Conclusion: In low-immunological-risk KTRs, no induction therapy achieves comparable AR prevention and renal function outcomes to BSX while reducing infection and hematological complications. These findings challenge the necessity of universal induction therapy in this population and support a personalized approach to immunosuppression protocols
