# Supplementary figures and images for: Evaluating the efficacy of basiliximab versus no induction in low-immunological-risk kidney transplant recipients: a propensity score matched analysis
Source: Ren Fail. 2025 Feb 20;47(1):2460729. doi: 10.1080/0886022X.2025.2460729 (PMC11843659; doi:10.1080/0886022X.2025.2460729)

**Figure S3** Compare the triple maintenance regimen among the two groups Before PSM

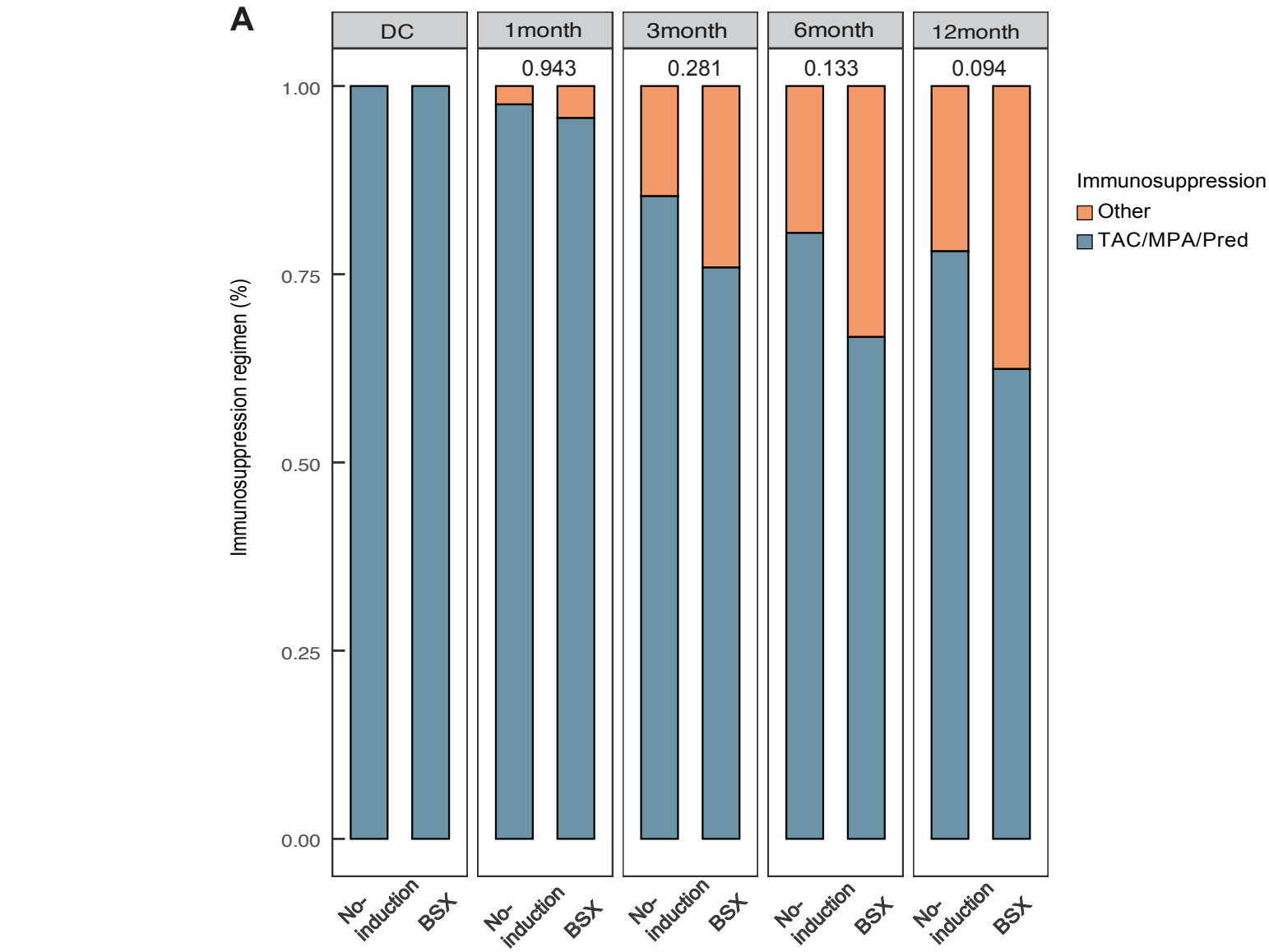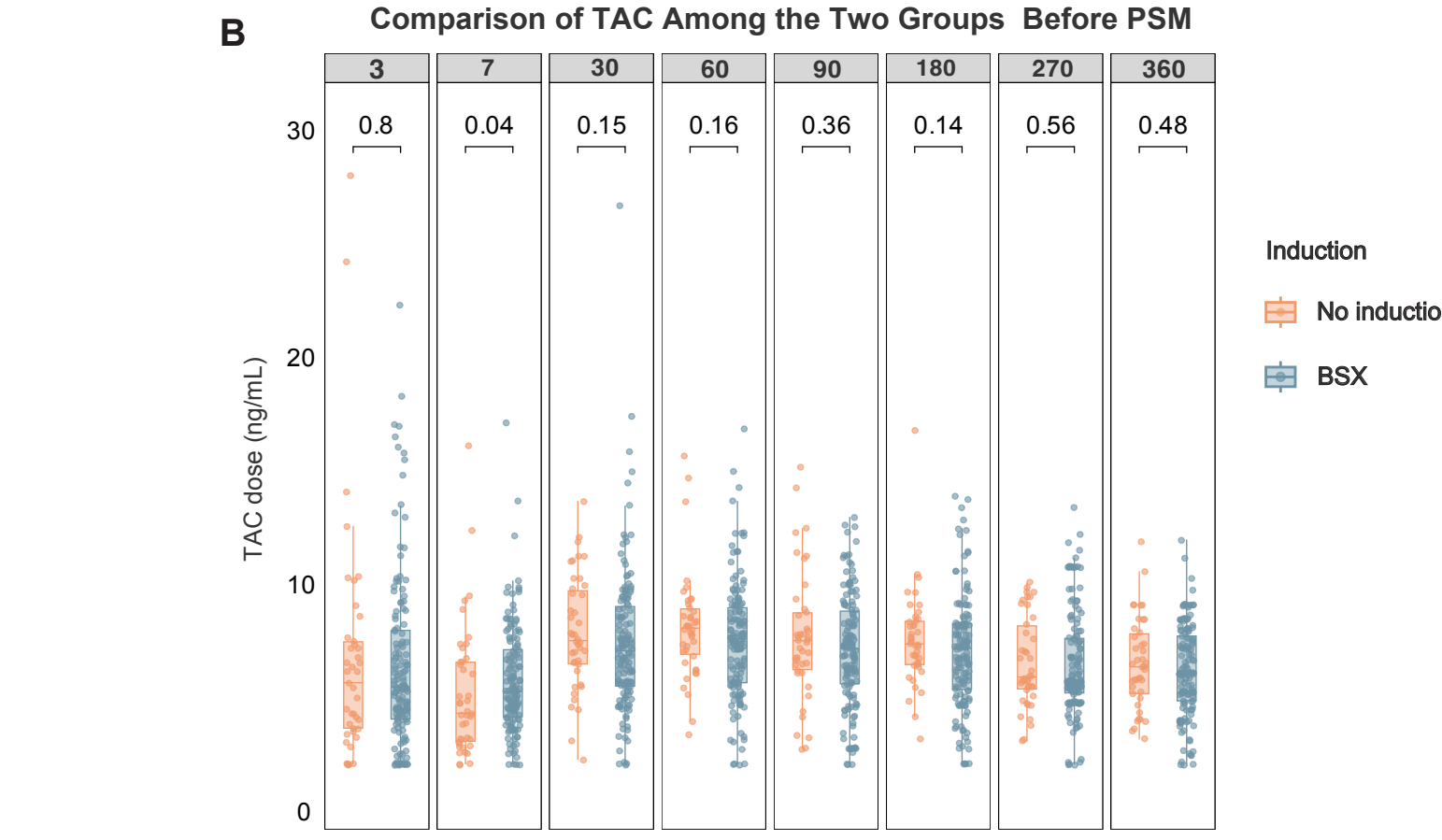

Supplement: Figure S3.pdf [file IRNF_A_2460729_SM4955.pdf]

Fig.1

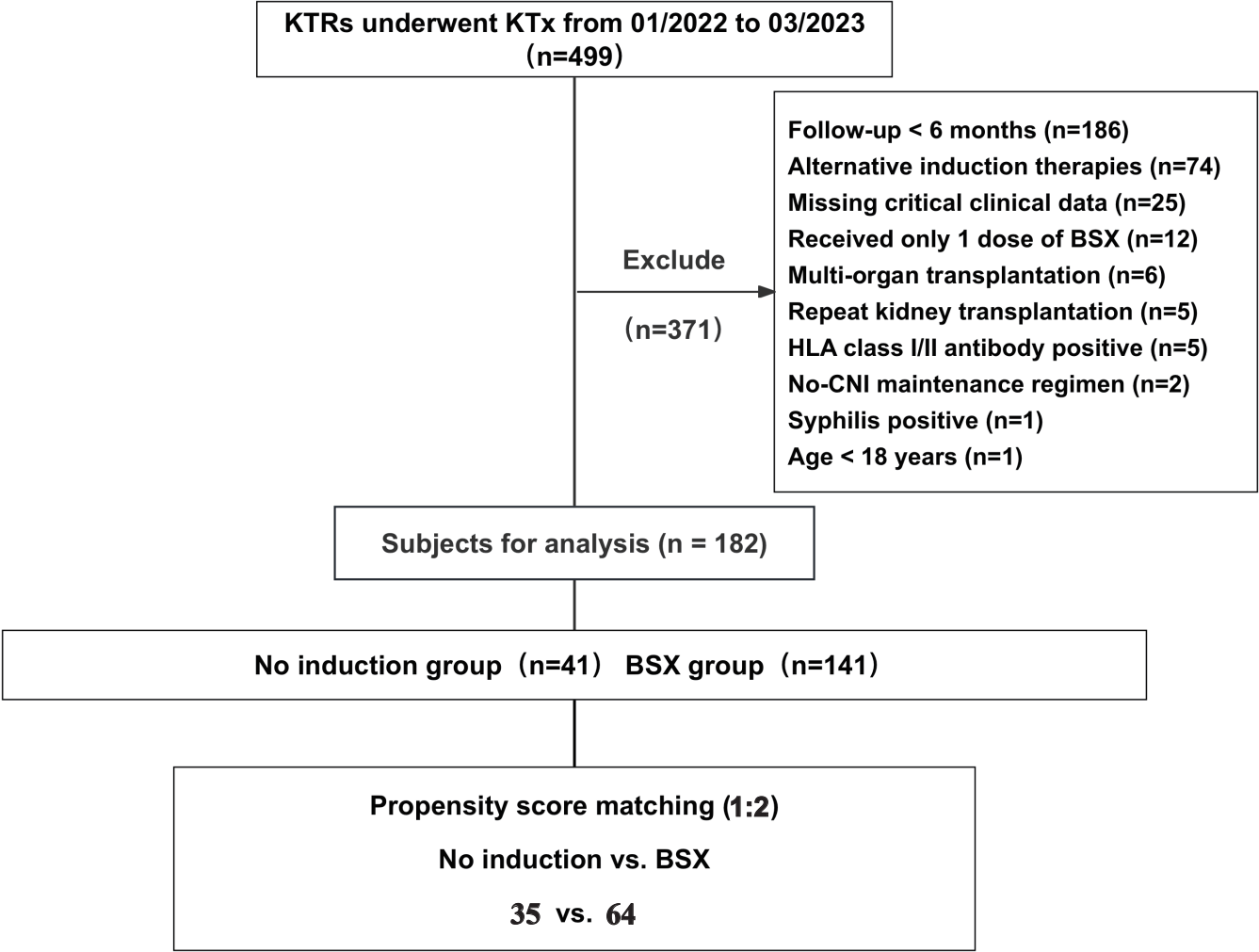

Supplement: Figure 1.pdf [file IRNF_A_2460729_SM4952.pdf]

Figure 2

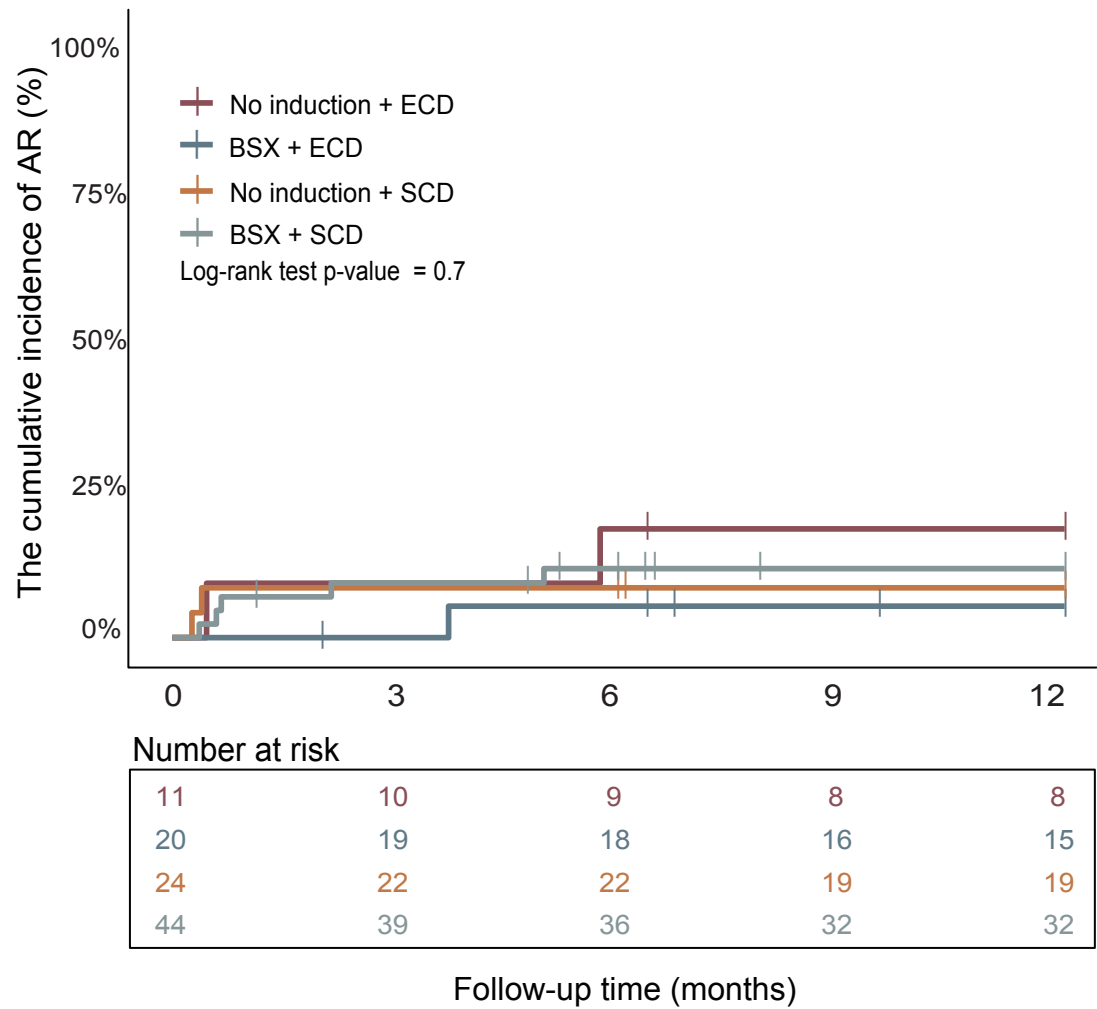

Supplement: Figure 2.pdf [file IRNF_A_2460729_SM4949.pdf]

Figure S2

A

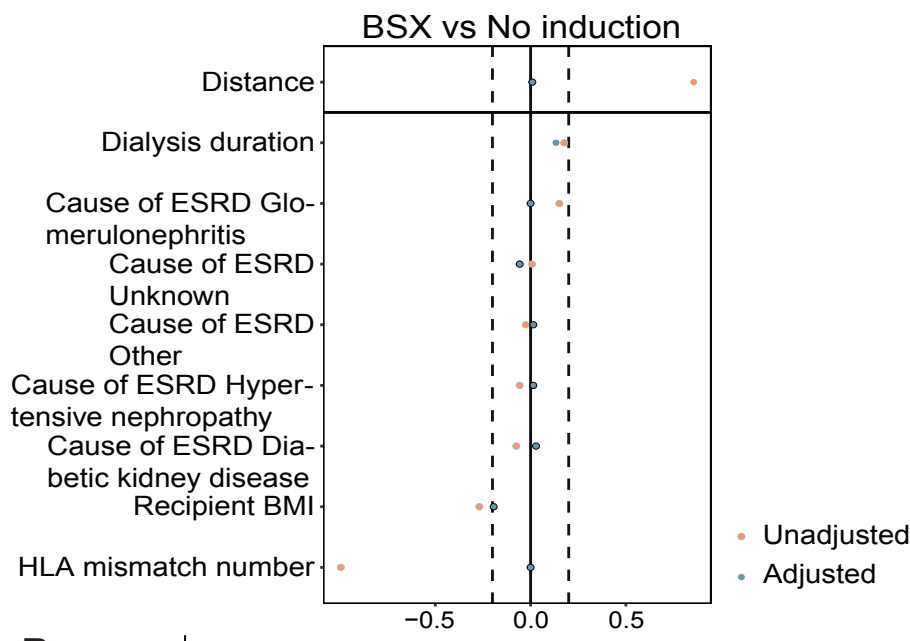

B

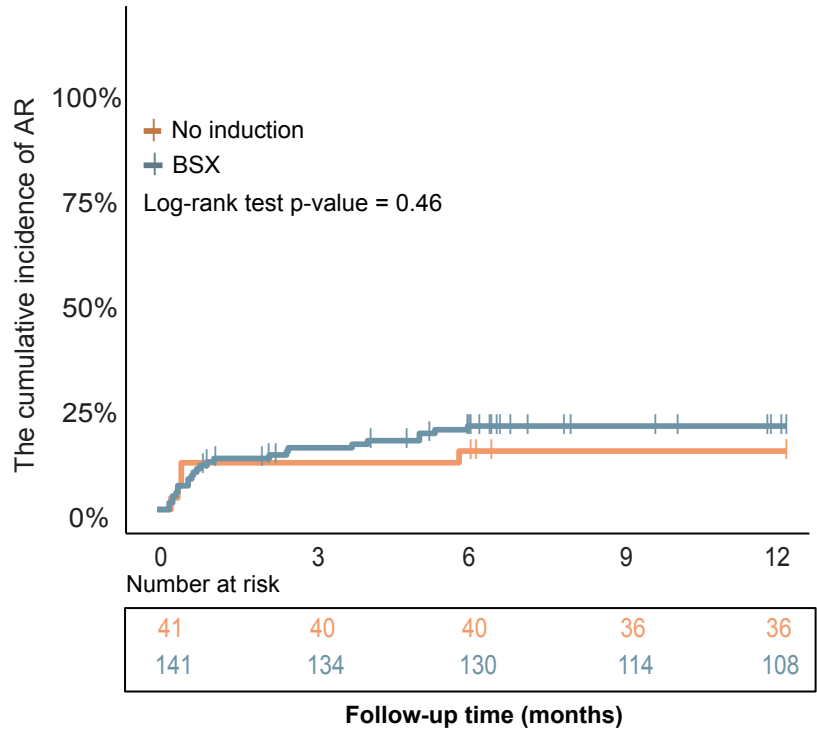

C

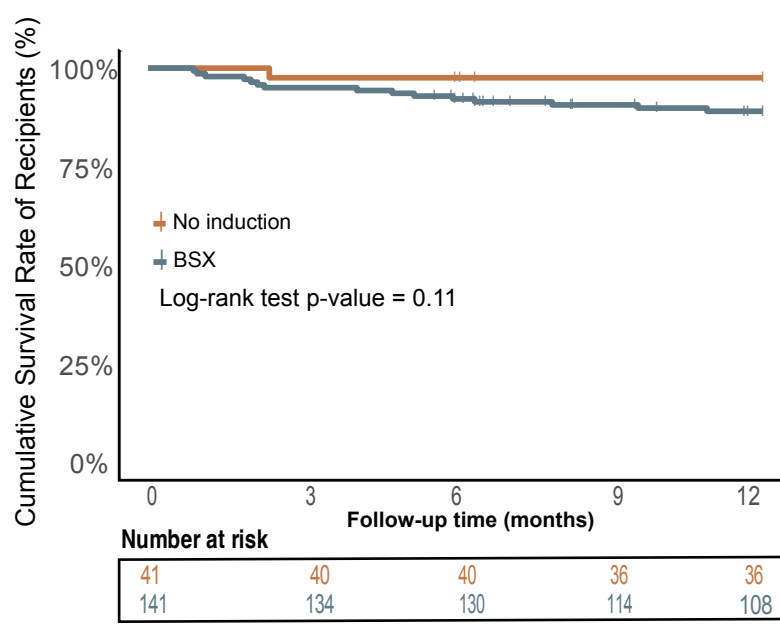

D

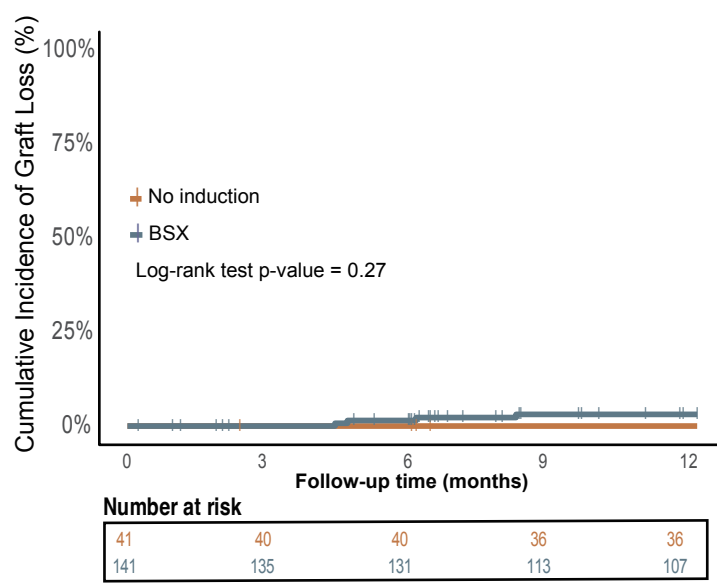

Supplement: Figure S2.pdf [file IRNF_A_2460729_SM4948.pdf]
