# Supplementary material for: Evaluating the efficacy of basiliximab versus no induction in low-immunological-risk kidney transplant recipients: a propensity score matched analysis
Source: Ren Fail. 2025 Feb 20;47(1):2460729. doi: 10.1080/0886022X.2025.2460729 (PMC11843659; doi:10.1080/0886022X.2025.2460729)
Supplement: Supplement Material.docx [file IRNF_A_2460729_SM4950.docx]

Table S1 : SMD Values Before and After PSM Matching: No Induction vs. BSX

| Variable | SMD_Before | SMD_After |
| --- | --- | --- |
| Recipient _Age | 0.001956084 | 0.18678855 |
| Recipient _BMI | -0.253422329 | -0.18860728 |
| Recipient _Gender_male | 0.026466009 | 0.05714286 |
| HLA_mismatch_number | -0.828252775 | 0 |
| Dialysis_duration | 0.18636094 | 0.13134211 |
| Cause_of_ESRD_Diabetic_kidney_disease | -0.074900536 | 0.02857143 |
| Cause_of_ESRD_Glomerulonephritis | 0.150838955 | 0 |
| Cause_of_ESRD_Hypertensive_nephropathy | -0.057602491 | 0.01428571 |
| Cause_of_ESRD_Other | -0.025255146 | 0.01428571 |
| Cause_of_ESRD_Unknown | 0.006919218 | -0.05714286 |
| Dialysis_type_Hemodialysis | 0.030963501 | 0.02857143 |
| Dialysis_type_Peritoneal_dialysis | 0.01158969 | 0.01428571 |
| Dialysis_type_Preemptive_transplantation | -0.042553191 | -0.04285714 |
| Donor _Age | -0.033084657 | 0.01196134 |
| Donor _BMI | -0.051367689 | 0.06389462 |
| Donor _Gender_male | -0.049299429 | -0.04285714 |
| Donor_AKI_No_AKI | 0.050856253 | 0.05714286 |
| Donor _Complication_no | -0.01522228 | -0.07142857 |
| Donor_type_SCD | -0.040477426 | -0.01428571 |

Table S2. Comparison of Total Methylprednisolone Dose Between No Induction and BSX Groups Before PSM

| Variable | No induction （n=41） | BSX （n=141） | P* |
| --- | --- | --- | --- |
| Dose(mg/kg), Median [IQR] | 35.1 [30.8;39.2] | 32.5 [28.6;37.7] | 0.117 |
| *Mann-Whitney U test | | | |

| Table S3. Comparison of Clinical Characteristics Between No induction and BSX Groups  After PSM | | | | |  |
| --- | --- | --- | --- | --- | --- |
|  |  |  |  |  |  |
| Variable | Total cohort （n=99） | No induction （n=35） | BSX （n=64） | P* |  |
| Recipient Age (yr), Median [IQR] | 36.0[31.0;46.0] | 41.0[31.0;47.0] | 35.0[31.0;43.0] | 0.362 |  |
| Recipient BMI (kg/m2), Median [IQR] | 21.6[19.0;23.7] | 21.3[19.4;23.4] | 21.7[18.9;24.0] | 0.52 |  |
| Recipient Gender, n (%) |  |  |  | 0.852 |  |
| female | 28(28.3%) | 9(25.7%) | 19(29.7%) |  |  |
| male | 71(71.7%) | 26(74.3%) | 45(70.3%) |  |  |
| HLA mismatch number, Median [IQR] | 3.00[3.00;3.00] | 3.00[2.00;3.00] | 3.00[3.00;3.00] | 0.737 |  |
| Cause of ESRD, n (%) |  |  |  | 0.593 |  |
| Diabetic kidney disease | 1(1.01%) | 1(2.86%) | 0(0.00%) |  |  |
| Glomerulonephritis | 20(20.2%) | 8(22.9%) | 12(18.8%) |  |  |
| Hypertensive nephropathy | 5(5.05%) | 2(5.71%) | 3(4.69%) |  |  |
| Other | 2(2.02%) | 1(2.86%) | 1(1.56%) |  |  |
| Unknown | 71(71.7%) | 23(65.7%) | 48(75.0%) |  |  |
| Dialysis type, n (%) |  |  |  | 0.8 |  |
| Hemodialysis | 81(81.8%) | 29(82.9%) | 52(81.2%) |  |  |
| Peritoneal dialysis | 16(16.2%) | 6(17.1%) | 10(15.6%) |  |  |
| Preemptive transplantation | 2(2.02%) | 0(0.00%) | 2(3.12%) |  |  |
| Dialysis duration (month), Median [IQR] | 21.0[12.5;36.0] | 24.0[14.0;39.5] | 20.0[12.0;36.0] | 0.543 |  |
| Donor Age (yr), Median [IQR] | 46.0[37.0;58.0] | 48.0[41.0;55.5] | 46.0[35.8;59.8] | 0.892 |  |
| Donor BMI (kg/m^2^), Median [IQR] | 22.9[20.5;25.2] | 23.9[21.0;24.9] | 22.9[20.3;25.1] | 0.784 |  |
| Donor Gender, n (%) |  |  |  | 0.822 |  |
| female | 20(20.2%) | 8(22.9%) | 12(18.8%) |  |  |
| male | 79(79.8%) | 27(77.1%) | 52(81.2%) |  |  |
| Donor AKI, n (%) |  |  |  | 0.753 |  |
| AKI | 23(23.2%) | 7(20.0%) | 16(25.0%) |  |  |
| No AKI | 76(76.8%) | 28(80.0%) | 48(75.0%) |  |  |
| D Complication, n (%) | 33(33.3%) | 13(37.1%) | 20(31.2%) | 0.71 |  |
| Donor type, n (%) |  |  |  | 1 |  |
| ECD | 31(31.3%) | 11(31.4%) | 20(31.2%) |  |  |
| SCD | 68(68.7%) | 24(68.6%) | 44(68.8%) |  |  |
| *Mann-Whitney U test and Chi-square test | | | | |  |

| Table S4. The Cumulative Incidence of First Acute Rejection Within 12 Months in the Two groups Before PSM | | | | | | | |
| --- | --- | --- | --- | --- | --- | --- | --- |
| Groups | Months | n.risk | n.event | Cumulative Event Rate | Std.Err | Lower 95% CI | Upper 95% CI |
| No induction | 3 | 37 | 4 | 0.098 | 0.0463 | 0.002 | 0.184 |
|  | 6 | 36 | 1 | 0.122 | 0.0511 | 0.016 | 0.217 |
|  | 12 | 32 | 0 | 0.122 | 0.0511 | 0.016 | 0.217 |
| BSX | 3 | 117 | 18 | 0.129 | 0.0283 | 0.071 | 0.183 |
|  | 6 | 109 | 6 | 0.174 | 0.0324 | 0.108 | 0.235 |
|  | 12 | 91 | 0 | 0.174 | 0.0324 | 0.108 | 0.235 |
| Overall | 3 | 154 | 22 | 0.122 | 0.0243 | 0.073 | 0.168 |
|  | 6 | 145 | 7 | 0.162 | 0.0276 | 0.106 | 0.214 |
|  | 12 | 123 | 0 | 0.162 | 0.0276 | 0.106 | 0.214 |
|  | | | | | | | |

Table S5. Postoperative Deaths and Graft Failure Within 12 Months Before PSM

| Variable | Total cohort （n=182） | No induction (n=41) | BSX (n=141) | p* |
| --- | --- | --- | --- | --- |
| Recipient deaths, n (%) | 20(8.85%) | 1(2.44%) | 15(10.6%) | 0.377 |
| Severe pneumonia | 14 (7.69%) | 1(2.44%) | 14(9.93%) |  |
| Cerebral hemorrhage | 1 (0.55%) | 0(0.00%) | 1(0.71%) |  |
| Allograft loss, n (%) | 7(3.08%) | 0(0.00%) | 4(2.84%) | 1.000 |
| TCMR | 1 (0.55%) | 0(0.00%) | 1(0.71%) |  |
| ABMR | 1 (0.55%) | 0(0.00%) | 1(0.71%) |  |
| FSGS | 1 (0.55%) | 0(0.00%) | 1(0.71%) |  |
| Unknow | 1 (0.55%) | 0(0.00%) | 1(0.71%) |  |
| FSGS：focal segmental glomerulosclerosis； *Mann-Whitney U test and Chi-square test | | | | |

Table S6. DGF Within 12 Months Before PSM

| Variable | Total cohort (n=182) | No induction (n=41) | BSX (n=141) | P* |
| --- | --- | --- | --- | --- |
| DGF, n (%) | 33 (18.1%) | 4(9.76%) | 29 (20.6%) | 0.177 |
| DGF Duration (days), Median [IQR] | 15.0 [8.50;20.0] | 12.0[10.5;15.0] | 15.0[7.75;21.8] | 0.867 |
| Lowest Creatinine (μmol/L), Median [IQR] | 162 [124;208] | 152[150;157] | 164[120;210] | 0.640 |
| Highest Creatinine (μmol/L), Mean (SD) | 1013 (193) | 1065(120) | 1007(200) | 0.514 |
| DGF Duration, Time from the first dialysis to the last dialysis during the DGF period. Lowest Creatinine, the lowest serum creatinine level recorded during the DGF period. Highest Creatinine, the highest serum creatinine level recorded during the DGF period. *Mann-Whitney U test and Chi-square test | | | | |

| Table S7. Multivariate Cox Regression Model After PSM | | | | |  |
| --- | --- | --- | --- | --- | --- |
|  | HR (95%CI), P-value | HR (95%Cl), P-value | HR（95%Cl）,P-value | HR (95%Cl), P-value |  |
| Group | Model 1 | Model 2 | Model 3 | Model 4 |  |
| No induction | Reference | Reference | Reference | Reference |  |
| BSX | 1.81  (0.23-2.88), 0.748 | 1.02  (0.25-4.08), 0.982 | 0.57  (0.16-2.07), 0.400 | 1.35  (0.37-4.88), 0.650 |  |
| **Model 1.** Unadjusted. **Model 2.** Adjusted for key baseline characteristics, including donor gender, donor age, donor BMI, donor creatinine, AKI donor , donor complications, and donor type. **Model 3.** Adjusted for key baseline characteristics, including recipient gender, recipient age, recipient BMI, HLA mismatch number, cause of ESRD, dialysis type, dialysis duration. **Model 4.** Adjusted for key baseline characteristics, including recipient gender, recipient age, recipient BMI, HLA mismatch number, cause of ESRD, dialysis type, dialysis duration, donor gender, donor age, donor BMI, donor creatinine, AKI donor , donor complications, and donor type. | | | | |  |
|  |  |  |  |  |  |
|  |  |  |  |  |  |
|  |  |  |  |  |  |
|  |  |  |  |  |  |
|  |  |  |  |  |  |
|  |  |  |  |  |  |
|  |  |  |  |  |  |
|  |  |  |  |  |  |
|  |  |  |  |  |  |
