# Supplementary material for: Evaluating the efficacy of basiliximab versus no induction in low-immunological-risk kidney transplant recipients: a propensity score matched analysis
Source: Ren Fail. 2025 Feb 20;47(1):2460729. doi: 10.1080/0886022X.2025.2460729 (PMC11843659; doi:10.1080/0886022X.2025.2460729)
Supplement: Figure 3.pdf [file IRNF_A_2460729_SM4947.pdf]

Figure 3

A

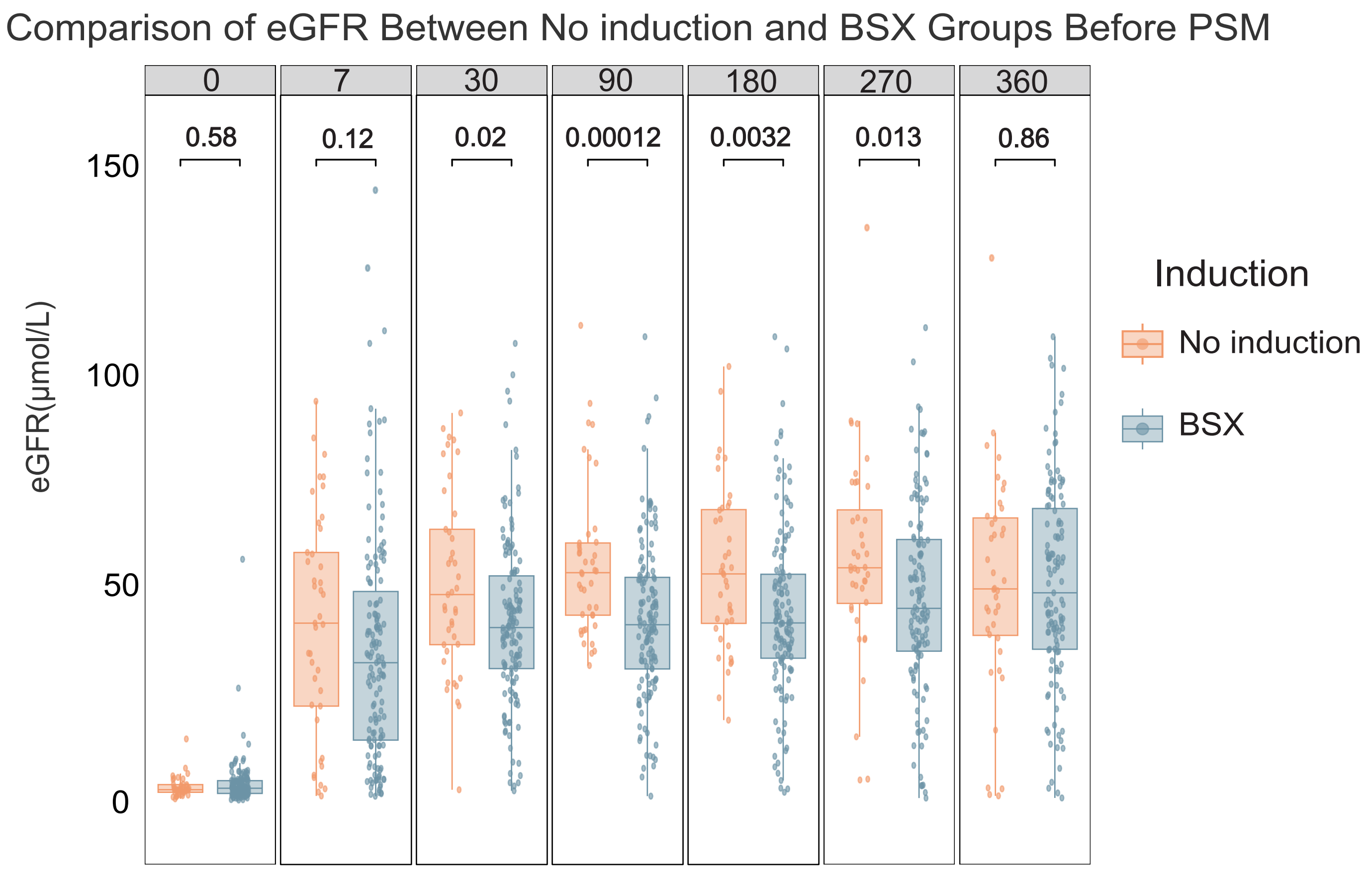

Comparison of eGFR Between No induction and BSX Groups Before PSM
0
7
30
90
180
270
360
0.58
0.12
0.02
0.00012
0.0032
0.013
0.86
eGFR(μmol/L)
150
100
50
0
Induction
No induction
BSX

B

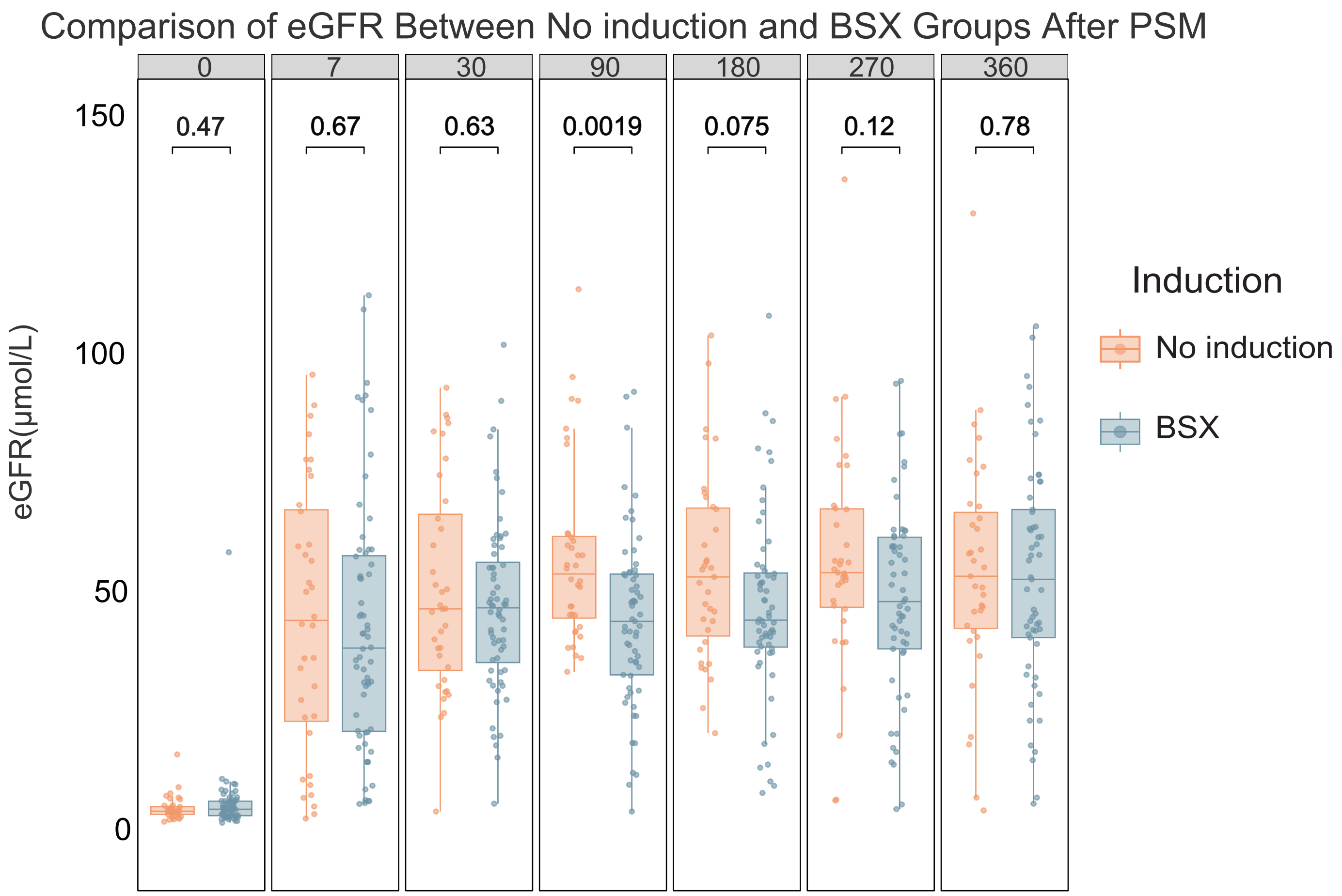

Comparison of eGFR Between No induction and BSX Groups After PSM
0
7
30
90
180
270
360
0.47
0.67
0.63
0.0019
0.075
0.12
0.78
eGFR(μmol/L)
150
100
50
0
Induction
No induction
BSX
